# Currents Induced by Injected Charge in Junction Detectors

**DOI:** 10.3390/s130912295

**Published:** 2013-09-12

**Authors:** Eugenijus Gaubas, Tomas Ceponis, Vidas Kalesinskas

**Affiliations:** Institute of Applied Research, Vilnius University, Sauletekio av. 9-III, LT-10223, Vilnius, Lithuania; E-Mails: tomas.ceponis@ff.vu.lt (T.C.); vidas.kalesinskas@ff.vu.lt (V.K.)

**Keywords:** photo-detectors, particle-detectors, Ramo's current, injected charge drift current

## Abstract

The problem of drifting charge-induced currents is considered in order to predict the pulsed operational characteristics in photo- and particle-detectors with a junction controlled active area. The direct analysis of the field changes induced by drifting charge in the abrupt junction devices with a plane-parallel geometry of finite area electrodes is presented. The problem is solved using the one-dimensional approach. The models of the formation of the induced pulsed currents have been analyzed for the regimes of partial and full depletion. The obtained solutions for the current density contain expressions of a velocity field dependence on the applied voltage, location of the injected surface charge domain and carrier capture parameters. The drift component of this current coincides with Ramo's expression. It has been illustrated, that the synchronous action of carrier drift, trapping, generation and diffusion can lead to a vast variety of possible current pulse waveforms. Experimental illustrations of the current pulse variations determined by either the rather small or large carrier density within the photo-injected charge domain are presented, based on a study of Si detectors.

## 1. Introduction

The drifting charge-induced current is often a prevailing component in detector signals. An analysis of this injected charge drift current is employed for the detection of photons or particles and for reconstruction of the electric field distribution over the charge drift area in detectors. However, the problem of the charge-induced currents remains a sophisticated issue in more complicated situations of photo- and particle-detectors when devices operate in partial or full depletion regimes with a complex field distribution in the presence of carrier capture-generation. Then, the induced current due to the charge trapping/de-trapping should be considered. The effects of the acting electric field screening or the gain caused by the interplay of the injected carrier charge and the bulk charge of ions in the depletion volume should also be involved. As usual, the principles of interpretation of the current transients in detectors under injected charge are based on the Shockley-Ramo theorem [1,2]. Ramo's theorem was derived based on consideration of Green's theorem and Gauss's law, thus, on the balance of electrostatic energy changes and on the analysis of bulk as well as of surface charge induced electrostatic fields. Ramo's current is derived at the assumption of the symmetry of an infinite electrode and of the spherical invariance of the potential surrounding an elementary charge. Therefore, the electrical capacitance of a real system of the finite surface area electrodes and its dependence on an applied voltage are out of consideration. Several attempts to generalize a simple Ramo's approach to multi-electrode and arbitrary space-charge field distribution systems had been made [3–6], although these solutions [3–13] are under debate, as referenced in [3–10]. As pointed out in [7], a simple Ramo's relation Equation (19) in [7] between the current within an external circuit and an introduced weighting field of geometrical sense is held only in the special case of moving charge in active area of a detector with a fixed external voltage applied. Therefore, a simple generalization or direct application of Ramo's theorem can be erroneous [7]. Most of the generalization approaches are again based on a consideration of electrostatic energy conservation [7–10]. For the space charge systems, like a pn junction and pin semiconductor devices, the simple Ramo's relations are not valid, and differential weighting potentials [8] are artificially introduced to restore the simple Ramo's type of expressions of the convection current. More systematic approaches are employed in [9,10], where space charge and different types of charges are included. However, the solutions obtained [9,10] for the estimation of the induced current on an arbitrary electrode provide no practical relevance of application with regard to semiconductor devices, as pointed out in [10], when the real velocity field of the charge domain is not known, and it should be evaluated by means of the complete analysis of field distribution. Thereby, an additional kinetic equation should be considered to determine the instantaneous velocity of the charge domain. However, applications of Ramo's theorem generally ignore this consideration, rendering the expressions obtained not practically applicable. The current pulse shape and duration are directly dependent on the temporal variation of the injected charge domain position within the inter-electrode space, where the instantaneous velocity and acceleration can be changeable. More complications appear within the consideration of a kinetic equation, when the injected charge amount can vary due to carrier traps inside the inter-electrode space.

In this work, the fixed area abrupt junctions with a plane-parallel geometry of electrodes are studied by considering the partial and over full-depletion regimes. The vectorial nature of the employed quantities of the surface charge domain, of the electric field and of the charge drift velocity is always kept in mind. The scalar relations are analyzed along the unite ortho-vector with proper signs ascribed to directions. To simplify the understanding of applied models, the single type of induced surface charge domain motion is initially considered. This simplification can be sufficient to model current transients due to highly absorbed photons or alpha-particles in Si detectors, while the generalization for a bipolar charge domain is also discussed. The specific features of the induced charge domain drift currents (ICDC) are revealed within analysis of the simulated ICDC transients and highlighted in illustrations of the experimental characteristics, measured on Si pin diodes.

## 2. Modelling of the Current Transients Induced by Injected Charge Drift

### 2.1. Geometry, Circuit and Electric Field Distribution for Different Regimes

**Figure 1.**
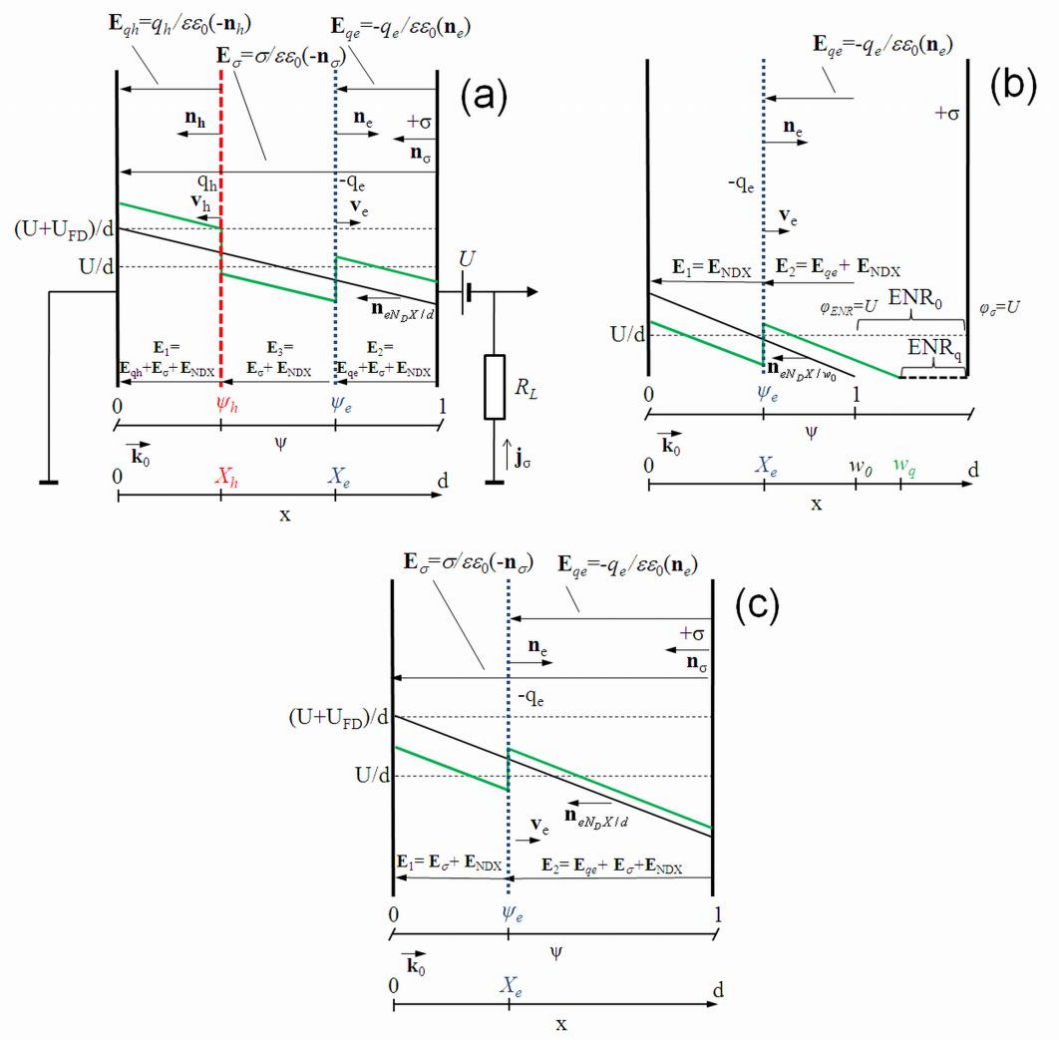
(**a**) Sketch of circuit for analysis of ICDC transients. Symbols denote as follows: *X_e_*,*_h_* is the instantaneous position of the drifting one-sided surface charge domain of the electrons (–*q_e_*) or holes (*q_h_*) (with normal vectors n*_e_* and n*_h_*), respectively; E_1_ = E*_qh_* + E*_σ_* + E*_NDX_* is the electric field caused by the superposition of the hole charge (*q_h_*) domain, the surface (of an area *S*) charge (+*σ*, with a normal vector n*_σ_*) on the high potential electrode and the local surface charge introduced to represent the electric field at *x* created by a bar (of a width (*d−x*)) of the bulk ion charge (with a normal vector n*_eNDX_*); E_2_ = E*_qe_* + E*_σ_* + E*_NDX_* is the electric field caused by the superposition of the electron charge (–*q_e_*) domain, the surface charge (+*σ*) and the local surface charge of the ion bar; E_3_ = E*_σ_* + E*_NDX_* is the superposition of field between the electron and hole domains and the surface charge of the ion bar; v*_e,h_* is the instantaneous velocity vector of a drift of the surface charge domain –*q_e_* or *q_h_*, respectively; *d* is the inter-electrode distance; *ε* and *ε_0_* are the material and vacuum dielectric permittivity, respectively; k_0_ is the unit ortho-vector in the uni-directional coordinate system; *ψ_h_* and *ψ_e_* are the dimensionless, normalized positions of the drifting hole or electron domains, respectively; **j***_σ_* is the current density; *U* is the external source of voltage; *R_L_* is the load resistor. The sketches are illustrated for the electric field instantaneous distribution due to the injected negative charge surface domain within a partially depleted (**b**) and fully depleted junction (**c**), respectively. *w_0_* is the steady-state depletion width; *w_q_(t)* is the charge drift introduced depletion width.

The same electrical circuit as in Ramo's theorem derivation, is considered: one electrode is grounded and the high potential is kept on the other one. These two electrodes are connected to the external voltage source in series with a load resistor to register a current transient within external circuit, as illustrated in Figure 1a. The circuit for detection of current transient *i(t)* routinely includes a load resistor and closed input of an oscilloscope type instrument. It is, as usual, assumed that the load resistor *R_L_* is properly chosen to get the recordable signal, and that a voltage drop on load resistor (*iR_L_*) for all the range of detected currents can be ignored, *i.e.*, *iR_L_* ≪ *U*.

As usual, a quasi-neutral domain of excess carriers is initially generated in particle detectors. It is accepted the domains are flat surface vector quantities. These domains injected by light or ionizing radiation are characterized by the surface charge density and the direction (vector) of the surface normal. Thereby, these domains are directly represented by the electric field of the surface charge. The sign (polarity) of the injected charge and the direction of the drift velocity vector are also included. For the grounded circuit, the single-side surface charge (and field) is ascribed to the voltage source. The drifting domain is also considered as the one-side surface charge vector correlated with drift velocity vector direction. The surface charge electric field vectors are initially considered, like the first Poisson equation. Then, the scalar equations for an instantaneous field distribution are analyzed. By applying an external field source, the injected carriers (by light or ionizing radiation) can be separated into oppositely moving surface charge sub-domains *q_e_* and *q_h_*, which induce charges of opposite sign on electrodes. The positive charge on the grounded electrode induced by a drifting charge domain is moved by the external source (battery) to the electrode of the high potential and vice versa. The latter charge transfer current is actually measured within the external circuit as a signal of either the charge domain drift or the charge density change. A sketch of the instantaneous field components is presented in Figure 1.

External voltage source *U* produces a positive surface charge *σ* on the high potential electrode, which is positioned at the distance *d* from the grounded electrode. Specific for the junction type detectors, the mobile charges within an active volume of a device lead to varied depletion width *w_0_* dependent on the applied (reverse *U_r_*) voltage on electrode. The grounded electrode is assumed to be located at the beginning of the coordinate system (*x* = 0). The complete neutralization of the depletion charge (e.g., *eN_D_*^+^) in the n-base active layer is obtained through respective depletion (*w_p_*_+_*)* of the other layer (*eN_A_*^−^) of a junction: *eN_D_*^+^*w_0_* − *eN_A_^-^w_p_*_+_ = 0. In the abrupt junction of a pin type diode, it is valid *w_p_*_+_ ≪ *w_0_*. Therefore, the assumption of the grounded electrode location at *x* = 0 is valid with precision of *w_p_*_+_*/w_0_* ≪ 1. The injection of the electron domain, with surface charge density *q_e_*, into the active volume of a detector at the instantaneous position *X_0_* causes a change of the charge on the high potential electrode, in the case of the over full-depletion regime.

For the partial depletion regime, this leads to changes of the depletion width *w_q_*, due to the mobile carriers in the electrically neutral region (ENR). As the electrodes are only connected to the external measurement circuit, the current induced by the moving charge –*q_e_* within the external circuit is determined by the surface charge *dσ* changes (being a full differential) in time: *dσ/dt*. To find these changes, the superposition of the induced fields should be considered.

The junction based detectors contain rather complicated field distribution, and, thereby, need specific analysis. The instantaneous field distribution in n-type conductivity region of the p^+^n abrupt junction structure during the monopolar drift of a negative charge in partially and over-depleted base region is sketched in Figures 1b, c, respectively.

### 2.2. Current Transient Induced by Charge Drift in Abrupt Junction

The analysis can be easily applied to the consideration of the barrier capacitance changes for unit area *C_b_* = *εε_0_/w_q_(t)*. The charge drift dependent depletion width *w_q_(t)* should then be introduced, due to the injected charge *q_e_*. The reverse biased steady-state depletion width *w_0_* = [2*εε_0_(U* + *U_bi_)/eN_Def_*] ^1/2^ serves as an equivalent of the inter-electrode spacing *d*. Here, *U_b_*_i_ denotes the built-in potential barrier, and *N_def_* is the effective dopant density. Variations of surface charge *σ* are equivalent to the changes of the surface charge at *w_0_*, induced by an additional depletion charge bar, as *σ* ∼ *eN_Def_(w_q_(t)* − *w_0_)*. It can be proved that consideration of the time dependent changes of the system dynamic capacitance *C_Sq_* (ascribed to a surface area unite) is equivalent to the analysis of the convection current. In semiconductor devices an important role is played by carrier capture and generation processes. Also, different regimes of the partial-depletion, of the full depletion and the over-depletion (depending on detector width and applied voltage) have their specific features. The mixed regimes of the electrode surface charge changes and of the depletion width variations are inherent for the applied voltage values close to the values *U_FD_* of the full depletion (*FD*). For clarity, these different regimes are separately analyzed below.

#### 2.2.1. Current Transients of the Injected Charge Drift in Partially Depleted Detector

This regime is partially discussed in [14]. Let's consider a regime of the applied reverse voltages *U_bi_* < *U* < *U_FD_* on the n-type conductivity layer at an assumption that the electron domain is injected nearby the metallurgic abrupt junction, and the strength of the electric field there is capable to separate the electron-hole pairs, with consequent extraction of holes into p^+^-type layer. This leads to a synchronous change of the depletion widths in the n- and p- type conductivity layers to keep the junction system electrically neutral behind the depletion *w_0,n_* and *w_p_*_+_ width boundaries. To simplify the analysis, an assumption of the asymmetric doping of n- and p-layers, *i.e.*, the abrupt p-i-n junction is accepted, which enables ones to ignore a voltage drop on p^+^-layer. It is also assumed that the external metallic electrodes are in a rapid dynamic balance with neutral n- and p^+^- layer material. Thus, a rate of the processes within an n-layer region is the slowest one. The latter processes determine the current transient caused by a drift of the injected electron domain.

Using the methodology described in [14], an instantaneous field distribution is obtained by integrating the first Poisson equation and by assuming an infinitesimally thin drifting domain of surface charge of density *q_e_*, as:

(1) $$E(x)=\{\begin{array}{c} E_{1}(x)=-(E_{1}(0)-\frac{eN_{\text{Def}}}{ɛ{ɛ}_{0}}x)=[\frac{eN_{\text{Def}}}{ɛ{ɛ}_{0}}(w_{q}-x)-\frac{q_{e}}{ɛ{ɛ}_{0}}],\text{for}\;x<X_{e}\\ E_{2}(x)=-(E_{1}(0)-\frac{eN_{\text{Def}}}{ɛ{ɛ}_{0}}x)-\frac{q_{e}}{ɛ{ɛ}_{0}}\equiv -\frac{eN_{\text{Def}}}{ɛ{ɛ}_{0}}(w_{q}-x),\text{for}\;x>X_{e}\\ E_{2}(X_{e})-E_{1}(X_{e})=-\frac{q_{e}}{ɛ{ɛ}_{0}},\text{for}\;x=X_{e} \end{array}$$

Here, a vector of the electric field is directed towards the junction, while a surface charge domain of electrons can drift towards high potential electrode. To find a depletion width *w_q_* and, alternatively, *E*_1_*(0)*, due to the injected charge *q_e_* domain and the external reverse bias voltage (*U_r_*) source, the second Poisson integral should be taken, which leads to expressions:

(2) $$w_{q}=\frac{ɛ{ɛ}_{0}}{eN_{\text{Def}}}(E_{1}(0)+\frac{q_{e}}{ɛ{ɛ}_{0}})=\sqrt{\frac{2ɛ{ɛ}_{0}}{eN_{\text{Def}}}[U+\frac{q_{e}}{ɛ{ɛ}_{0}}X_{e}]}=w_{0}\sqrt{\left(1+\frac{w_{0}^{2}\mu_{e}q_{e}/w_{0}}{\mu_{e}U_{r}ɛ{ɛ}_{0}}\frac{X_{e}}{w_{0}}\right)}=w_{0}\sqrt{1+\frac{\tau_{\text{TOF},w_{0}}}{\tau_{Mq,w_{0}}}\frac{X_{e}}{w_{0}}}$$

and:

(3) $$E_{1}(0)=\frac{eN_{\text{Def}}}{ɛ{ɛ}_{0}}\sqrt{\frac{2ɛ{ɛ}_{0}}{eN_{\text{Def}}}[U+\frac{q_{e}}{ɛ{ɛ}_{0}}X_{e}]}-\frac{q_{e}}{ɛ{ɛ}_{0}}$$

Here, a common depletion boundary condition (*E(w_q_)* = 0) and a proper root of the quadratic equation are accepted; *μ* denotes the carrier mobility. For the reverse biased junction, it is assumed as usual [15] that *U* = *U_r_*−*U_bi_*. Within a coordinate system at rest, the characteristic time parameters *τ_Mq,w0_* and *τ_TOF,w0_*, are defined relative to a steady-state width *w_0_*, instead of *d*. Thereby, the characteristic time parameters *τ_Mq,w0_* (time of the Maxwell relaxation of charge *q, Mq*) and *τ_TOF,w0_* (time of flight, *TOF*) are expressed as:

(4) $$\tau_{\text{TOF},w0}=\frac{w_{0}^{2}}{\mu_{e}U}$$

(5) $$\tau_{Mq,w0}\frac{ɛ{ɛ}_{0}}{\mu_{e}(q_{e}/w_{0})}$$

These characteristic times, namely, their equality (*τ_Mq,w0_* = *τ_TOF,w0_*), can be a measure for the validity of the electrostatic induction approach. These characteristic times implicate the response time of the extended (of distributed charge) electrode (*τ_TOF,w0_*) and of a drifting domain (*τ_Mq,w0_*).

A steady-state depletion width *w_0_* is expressed by a well-known formula derived within depletion approximation [15] as *w_0_* = *(2εε_0_U/eN_D_)*^1/2^. Consequently, a barrier capacitance is obtained as:

(6) $$C_{b,Sq}=\frac{ɛ{ɛ}_{0}}{w_{q}}\equiv \frac{ɛ{ɛ}_{0}}{w_{0}\sqrt{1+\frac{\tau_{\text{TOF},w_{0}}}{\tau_{Mq,w_{0}}}\frac{X_{e}}{w_{0}}}}=\frac{C_{b0}}{\sqrt{1+\frac{\tau_{\text{TOF},w_{0}}}{\tau_{Mq,w_{0}}}\frac{X_{e}}{w_{0}}}}$$

where *C_b0_* = *εε_0_/w_0_*. It can be noticed that *w_q_* > *w_0_*, and, therefore, the barrier capacitance decreases due to the injected charge domain.

It can be inferred from Equations (1–6), that the regime of the surface charge domain drift within a partially depleted layer of a junction, can only be considered under a few restrictions on relations among values of the applied voltage, the doping and the injected charge density. A spatial range for *w_q_* variations is limited by a geometrical width *d* of a n-base layer (and consequently by the barrier capacitance decrease to its geometrical capacitance value), as:

(7) $$w_{q}=\sqrt{\frac{2ɛ{ɛ}_{0}U}{eN_{\text{Def}}}[1+\frac{q_{e}w_{0}}{ɛ{ɛ}_{0}U}]}<d\sqrt{\frac{2ɛ{ɛ}_{0}U_{FD}}{eN_{\text{Def}}}}$$

For electrodes of surface unite area *S* = 1, this leads to the inequalities, written as:

(8) $$\frac{ɛ{ɛ}_{0}}{eN_{\text{Def}}d}\frac{q_{e}}{ɛ{ɛ}_{0}}\equiv \frac{\tau_{M,\text{Ndef}}}{\tau_{Mq}}<\frac{1}{2}\frac{d}{w_{0}}(1-\frac{U}{U_{FD}})$$

Here, *τ_M,Ndef_* = *εε_0_/eμ_e_N_Def_* is the material dielectric relaxation time within a space charge region. Inequality Equation (8) leads to a limitation:

(9) $$q_{e}<\frac{eN_{\text{Def}}d^{2}}{2w_{0}}(1-\frac{U}{U_{FD}})$$

of the injected surface charge which can be moved off by *U* < *U_FD_*. The current density is obtained by the analysis of the time dependent variations of a surface charge on electrode due to the extracted electrons, as:

(10) $$j=\frac{d\sigma }{dt}=U\frac{\partial C}{\partial X_{e}}\frac{\partial X_{e}}{\partial t}=U\frac{\partial C}{\partial w_{q}}\frac{\partial w_{q}}{\partial X_{e}}\frac{\partial X_{e}}{\partial t}$$

The rearranged (by these differentiation procedures) expression of a module of the current density of the injected charge domain (ICD) drift can be represented as follows:

(11) $$\left|j_{\text{ICD}}\right|=q_{e}\frac{1}{w_{0}}\frac{1}{2{\left(1+\frac{\tau_{\text{TOF},w_{0}}}{\tau_{Mq,w_{0}}}\frac{X_{e}}{w_{0}}\right)}^{3/2}}\frac{dX_{e}}{dt}=q_{e}\frac{1}{w_{0}}K_{\tau }\frac{dX_{e}}{dt}=q_{e}K_{t}\frac{d\psi_{w_{0}}}{dt}$$

The obtained scalar form of the current density within a coordinate system at rest (*w_0_*) is very similar to that of the Ramo's current expression. The main difference is an appearance of a coefficient *K_τ_*, dependent on the dimensionless position Ψ* = *X_e_/w_0_* of a drifting surface charge domain within *w*_0_, and it is composed of the characteristic times as:

(12) $$K_{\tau }=\frac{1}{2{\left(1+\frac{\tau_{\text{TOF},w_{0}}}{\tau_{Mq,w_{0}}}\psi^{*}\right)}^{3/2}}$$

The appearance of the coefficient *K_τ_* is a specific feature of the non-fixed position of the virtual electrode (*w_q_*), charge on which is varied by a changed depletion range of ions. Simultaneously, the possible drift length is also dependent on the rate of the formation of *w_0_* and *w_q_*, *i.e.*, on the characteristic time *τ_M,Ndef_* = *εε_0_/eμN_Def_* = *εε_0_/eμn_ENR_* of the stabilization of the transitional *λ*-thick layer (between the depletion and ENR layers) due to extraction of the mobile carriers *n_ENR_* = *N_Def_* from ENR. This transitional *λ* layer is related to the Debye screening length [15].

The additional scalar equation (with properly accepted vector direction sign) for a velocity of the charge domain drift is now expressed as follows:

(13) $$\frac{dX_{e}}{dt}=\mu_{e}E(X_{e})=-\mu_{e}E_{2}(X_{e})=\mu_{e}\frac{eN_{\text{Def}}}{ɛ{ɛ}_{0}}(w_{q}-X_{e})=\frac{w_{0}}{\tau_{M,\text{Ndef}}}(\sqrt{1+\frac{\tau_{\text{TOF},w0}}{\tau_{Mq,w0}}\frac{X_{e}}{w_{0}}}-\frac{X_{e}}{w_{0}})$$

The rearranged equation into the dimensionless Ψ* = *X_e_/w_0_* form can be written as:

(14) $$\frac{d\psi^{*}}{dt}=\frac{1}{\tau_{M,\text{Ndef}}}[\sqrt{1+\frac{\tau_{\text{TOF},w_{0}}}{\tau_{Mq,w_{0}}}\psi^{*}}-\psi^{*}]$$

with the adequate boundary conditions, as *t*=0 for Ψ*= Ψ_0_* and *t*=*t_dr_* for Ψ*= 1, respectively. This Equation (14) is the first-order ordinary non-linear equation, a solution of which is expressed as:

(15) $$t=\tau_{M,\text{Ndef}}[\int_{\psi_{0}^{*}}^{\psi^{*}(t)}\frac{1}{\sqrt{1+\xi \frac{\tau_{\text{TOF},w_{0}}}{\tau_{Mq,w_{0}}}}-\xi }d\xi ]$$

Extraction of the Ψ*(t) function, by integrating Equation (15), might be complicated [16], and it can commonly be found by a numerical solution. These solutions Ψ*(t) are only determined for an interval of the *ζ* values, evaluated by using condition [1 + *ζ(τ_TOF,w0_/τ_Mq,w0_)*] ^1/2^ − *ζ* > 0. The same difficulty appears in evaluation of drift time *t_dr_*, implemented by inserting the second boundary condition Ψ* = 1:

(16) $$t_{dr}=\tau_{M,\text{Ndef}}[\int_{\psi_{0}^{*}}^{1}\frac{1}{\sqrt{1+\xi \frac{\tau_{\text{TOF},w_{0}}}{\tau_{Mq,w_{0}}}}-\xi }d\xi ].$$

Then, the obtained Ψ*(t) should be inserted into the right hand side of Equations (11) and (12). Actually, a direct numerical solution of Equation (14) might be preferable in order to simulate the current density transients. The initial component of a rise to the pulse vertex (*j_ICD,F_(t)*) and the rearward relaxation component (*j_ICD,R_(t)*) of a current pulse can also be modelled. Here, it is assumed for simplicity that the kinetic equation of motion Equation (14) is only slightly modified due to τ*_Mq,w0_* during *q_e_* injection.

For *N_e_* carriers located within a domain on its surface area *S_e_*, comprising a surface density *q_e_* = *eN_e_/S_e_*, the injected charge drift is assumed to be a uni-directional process. Therefore, to relate more adequately the characteristic times *τ_TOF,w0_* and *τ_Mq,w0_*, the inequality Equation (9) could be rearranged as:

(17) $$\frac{\tau_{\text{TOF},w_{0}}}{\tau_{Mq,w_{0}}}<1$$

Thus, for the partially depleted junction, both components *d^2^/S_e_* < 1 and *(*1−*U/U_FD_)* < 1 should be small, and these conditions ensure that *τ_TOF,w0_/τ_Mq,w0_* < 1. Actually, the correlated drift of the injected charge domain (as assumed for the Ramo's regime) can only be implemented at *τ_TOF,w0_/τ_Mq,w0_* ≈ 1. Thereby, the exact Ramo's regime is impossible for the partially depleted junction if *K_τ_* ≠ 1.

Generally, variation of an initial component *j_ICD,F_(t)* as a function of time *t* within the current pulse evolution is described for the time interval 0 ≤ *t* ≤ *t_dr_* as:

(18) $$j_{\text{ICD},F}(t)=\frac{q_{e}}{2\tau_{M,N\text{d}ef}}\frac{\left[\sqrt{1+\frac{\tau_{\text{TOF},w_{0}}}{\tau_{Mq,w_{0}}}\psi^{*}(t)}-\psi^{*}(t)\right]}{{\left[1+\frac{\tau_{\text{TOF},w_{0}}}{\tau_{Mq,w_{0}}}\psi^{*}(t)\right]}^{3/2}}$$

**Figure 2.**
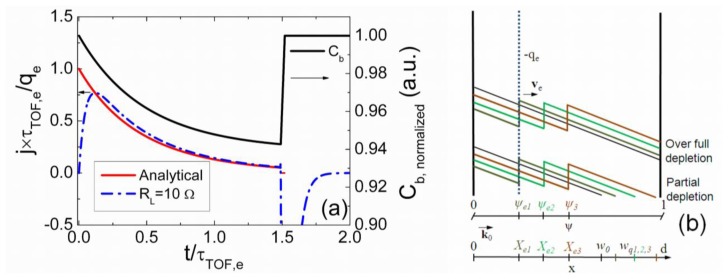
(**a**) Normalized current density *j* × *τ_TOF,e_/q_e_* transients simulated without including of the external circuit impact (red solid curve) and under the impact of external circuit with *R_L_* = 10 Ω (dash-dotted blue curve). Solid black curve represents the barrier capacitance variations during charge drift. (**b**) The electric field redistribution within the partially and the over-depleted diode layer during the drift of the electron domain.

Equation (18) describes a component of the pulse with a decreasing current shape within the pulse vertex, Figure 2.

The rearward component (*j_ICD,R_(t)*) (Figure 2) of the current pulse is determined by the processes of the system capacitance *C_b,Sq_* restore to its steady-state value *C_b,S0_* = εε_0_/*w_0_*. At arrival of the drifting charge domain to the *w_0_* location, the surface charge field *q_e_/εε_0_* is equal to the electrostatic induction charge field determined by a depletion charge bar *eN_D_(w_q_*−*w_0_)/εε_0_*. Therefore, the field *q_e_/εε_0_* is completely screened being at *w_0_*, as the reverse voltage determined field of positively charged ions becomes zero at the point *w_0_*. Thus, from the time instant *t* = *t_dr_*, an interplay of carrier diffusion from electrically neutral region (ENR) and surface charge field *q_e_/εε_0_* determines the reduction of the *w_q_* to the steady-state value *w_0_*. This process originates a current (relatively to a domain drift one) determined by narrowing of the depletion region, −carriers from the ENR drift into the opposite direction relative to the injected domain drift. This current represents the decreasing with time (*t*−*t_dr_*) component.

This current component may be responsible for the appearance of the offset within a current transient, inherent for the partially depleted detector. Duration of this process is determined by a dielectric relaxation time of the material, namely, *τ_M,Ndef_*. This current flows until the instant *t_Cw0_* of the barrier capacitance restore to its stationary value *εε_0_/w_0_*. It is determined by the barrier capacitance charging current. Superposition of these currents leads to a current relaxation component expressed as:

(19) $$j_{\text{ICD},R}(t)=j_{\text{ICD}}(t_{dr})\exp[\frac{\left(t-t_{dr}\right)}{\tau_{M,\text{Ndef}}}]$$

applied for the time scale of *t_dr_* < *t* ≤ *t_Cw0_*. Here, *C_w0_* = *C_b,S0_S*, including the surface area *S* of electrode.

As a result, the current density within a pulsed ICD transient is expressed as follows:

(20) $$j_{\text{ICD}}(t)=\{\begin{array}{c} j_{\text{ICD},F}(t)=\frac{q_{e}}{2\tau_{M,\text{Ndef}}}\frac{\left[\sqrt{1+\frac{\tau_{\text{TOF},w_{0}}}{\tau_{Mq,w_{0}}}\psi^{*}(t)}-\psi^{*}(t)\right]}{{\left[1+\frac{\tau_{\text{TOF},w_{0}}}{\tau_{Mq,w_{0}}}\psi^{*}(t)\right]}^{3/2}},\text{for}\;t\leq t_{dr}\\ j_{\text{ICD},R}(t)=j_{\text{ICD}}(t_{dr})\exp[\frac{\left(t-t_{dr}\right)}{\tau_{M,\text{Ndef}}}],\text{for}\;t_{dr}<t\leq t_{Cw0} \end{array}$$

The offset current relaxation to zero is additionally governed by the parameters of the external circuit. The relaxation component of the current pulse is determined by the relaxation processes within an RC chain consisting of the system capacitance *C_b,Sq_* and a load resistor *R_L_*. These elements together with the value of the applied external voltage determine the amplitude as well as the shape of the current pulse, and duration of the *j_ICD,F_* and *j_ICD,R_* components.

#### 2.2.2. Current Transients in Fully Depleted Detector

The surface charge on a metallic (or heavily doped layer) electrode changes together with the space charge bar width due to a moving surface charge domain, when external voltage *U* is equal to or exceeds the full depletion (*U_FD_*) value, *U ≥ U_FD_*. This happens due to a lack of semiconductor material (supporting mobile carriers) width (relative to a partially depleted layer case) to withstand the action of the electric field due to the injected charge. The equilibrium carriers are extracted into the external electrode, if depletion covers completely the active layer width.

Again, using the methodology described above, a field distribution for the negative drifting charge is obtained by taking the first Poisson equation:

(21) $$E(x)=\{\begin{array}{c} E_{1}(x)=-\frac{\sigma }{ɛ{ɛ}_{0}}-\frac{eN_{\text{Def}}d}{ɛ{ɛ}_{0}}(1-\frac{x}{d}),\text{for}\;x<X_{e}\\ \begin{array}{l} E_{2}(x)=-\frac{\sigma }{ɛ{ɛ}_{0}}-\frac{eN_{\text{Def}}d}{ɛ{ɛ}_{0}}(1-\frac{x}{d})-\frac{q_{e}}{ɛ{ɛ}_{0}},\text{for}\;x>X_{e}\\ E_{2}(X_{e})-E_{1}(X_{e})=-\frac{q_{e}}{ɛ{ɛ}_{0}},\text{for}\;x=X_{e} \end{array} \end{array}$$

and the second Poisson integral as:

(22) $$U=\frac{d}{ɛ{ɛ}_{0}}\sigma +\frac{eN_{\text{Def}}d^{2}}{2ɛ{ɛ}_{0}}+q_{e}\frac{d}{ɛ{ɛ}_{0}}(1-\frac{X_{e}}{d})$$

or, alternatively, including the value of *U_FD_* = *eN_Def_d^2^/*2*εε_0_*, it can be re-arranged as:

(23) $$U-U_{FD}=\frac{d}{ɛ{ɛ}_{0}}\sigma +q_{e}\frac{d}{ɛ{ɛ}_{0}}(1-\frac{X_{e}}{d})$$

The solution for a scalar surface charge density on the high potential electrode is expressed as either:

(24) $$\sigma =(U-U_{FD})\frac{ɛ{ɛ}_{0}}{d}-q_{e}(1-\frac{X_{e}}{d})=UC_{SN_{D}q}$$

or:

(25) $$\sigma =(U-U_{FD})\frac{ɛ{ɛ}_{0}}{d}-q_{e}(1-\frac{X_{e}}{d})=(U-U_{FD})C_{SN_{D}q}^{*}$$

with:

(26) $$C_{SN_{D}q}=\frac{ɛ{ɛ}_{0}}{d}(1-\frac{U_{FD}}{U})-\frac{q_{e}}{U}(1-\frac{X_{e}}{d})$$

or:

(27) $$C_{\text{SNq}}^{*}=\frac{ɛ{ɛ}_{0}}{d}-\frac{q_{e}}{\left(U-U_{FD}\right)}(1-\frac{X_{e}}{d})$$

The instantaneous field distribution can then be represented as follows:

(28) $$E(x)=\{\begin{array}{c} E_{1}(X_{e})=-\frac{U-U_{FD}}{d}+\frac{q_{e}}{ɛ{ɛ}_{0}}(1-\frac{X_{e}}{d})-\frac{eN_{\text{Def}}d}{ɛ{ɛ}_{0}}(1-\frac{X_{e}}{d}),\text{for}\;x<X_{e}\\ E_{2}(X_{e})=-\frac{U-U_{FD}}{d}-\frac{q_{e}}{ɛ{ɛ}_{0}}\frac{X_{e}}{d}-\frac{eN_{\text{Def}}d}{ɛ{ɛ}_{0}}(1-\frac{X_{e}}{d}),\text{for}\;x>X_{e} \end{array}$$

It can be deduced from Equations (26 and 27) that similarly to the above considered structures, a capacitance of the system is decreased relative to its steady-state value, due to a drifting (*X_e_(t)*) surface charge domain. It can be inferred a limitation for the charge density possible to move off:

(29) $$q_{e}<\frac{Uɛ{ɛ}_{0}}{d(1-\frac{X_{e}}{d})}[1-\frac{eN_{\text{Def}}d^{2}}{2ɛ{ɛ}_{0}U}]\equiv \frac{q_{C}}{\left(1-\frac{X_{e}}{d}\right)}[1-\frac{eN_{\text{Def}}d^{2}}{2ɛ{ɛ}_{0}U}]$$

This *q_e_* density also serves for evaluation of the relevant range of the positive capacitance *C_SNq_* values with *q_C_* = *UC_g_* = *Uεε_0_/d*.

A module of the current density, derived from Equations (26 and 27), in the case of the over full-depleted (OFD) junction, is again expressed as:

(30) $$j_{\text{OFD}}=\frac{d\sigma }{dt}=q_{e}\frac{1}{d}\frac{dX_{e}}{dt}$$

which formally represents the Ramo's type current component.

However, the considered situation for a fully-depleted junction is more complicated relative to those discussed above. The reason is a degenerated point *w_FD_* = *d*, Figure 2b. From one side, this is caused by the synchronous action of the capacitor-specific and the junction-inherent field distribution symmetries. As the junction determines a decreasing electric field shape, this field is exactly zero at the electrode (*w_FD_* ≡ *d*, the full-depletion condition) and *σ* does not change. Thus, any displacement current component for *w_FD_* = *d*, which is caused by the changes of an electric field behind the drifting domain, immediately becomes equal (transferred into) to a conductivity (convection) current, due to annihilation of the surface charge domain at *X_e_* = *d*. But the symmetry of a capacitor-inherent field implies that the displacement and convection currents, flowing within a capacitor in opposite directions, exactly compensate each other to keep the external voltage invariable. The displacement current in the over-depleted junction is obtained as:

(31) $$j_{\text{displ}}=ɛ{ɛ}_{0}\frac{\partial E}{\partial t}=\{\begin{array}{c} \left[-eN_{\text{Def}}d+q_{e}]\frac{1}{d}\frac{dX_{e}}{dt},\text{for}\;X_{e}<d/2,(A\right)\\ \left[en_{0}d+q_{e}]\frac{1}{d}\frac{dX_{e}}{dt},\text{for}\;X_{e}>d/2,(B\right) \end{array}$$

This *j_displ_* (Equation (31)) component is different (through an additional *eN_Def_dX_e_/dt* type term) from that *j_OFD_* derived by consideration of the changes of a surface charge on the electrode of the capacitor. Actually, the current (displacement and drift) components measured within external circuit cannot be separated, while the complete current is determined by the changes of charge on the external electrode.

On the other hand, the displacement current (Equation (31)) *eN_De_*_f_*dX_e_/dt* and the conduction current *en_0_dX_e_/dt* components completely compensate each other: the displacement current is caused by a space charge bar *eN_Def_Ψ_e_d* of ions, and the conduction current *en_0_dX_e_/dt* (Equation (31)) component, which appears due to a seeming extraction of equilibrium electrons *n_0_* and by producing the surface charge *en_0_d* on electrode, contain the opposite signs in Equations (31). This peculiarity occurs, if the space charge bar (*eN_Def_Ψ_e_d*) (either behind the injected surface charge domain (when *X_e_* = 0) or in front of it (when *X_e_* = *d*)) determines an appearance of the displacement current component to exactly compensate the conductivity current component *en_0_d* (to ensure invariance of external voltage *U*). Only for a singular set of boundary conditions *U* ≡ *U_FD_* and *X_e_* = 0 as well as *X_e_* = *d*, the displacement current *εε_0_∂E/∂t* is equal to *ρE* the material's conductivity current, *i.e.*, *εε_0_∂E/∂ t*= *ρE*, where *ρ* represents a conductivity of semiconductor material.

Thus, the complete current within an external circuit is again determined by the displacement current due to the injected charge, as obtained in Equation (30). While the space charge bar induced displacement currents (within Equation (31)) are employed to exactly compensate the conductivity (convection) current component in a fully depleted junction layer. These peculiarities should be kept in mind when considering the field of velocities to express definitely the time dependent current changes within a current pulse.

A dimensionless velocity field can be considered by using the accelerating electric field component for a geometric width *d* of the inter-electrode spacing as:

(32) $$\frac{v(X_{e})}{d}=\frac{d\psi }{dt}=\mu_{e}[\frac{U}{d^{2}}+\frac{1}{2}\frac{eN_{\text{Def}}}{ɛ{ɛ}_{0}}-\frac{eN_{\text{Def}}}{ɛ{ɛ}_{0}}\psi +\frac{q_{e}}{ɛ{ɛ}_{0}d}\psi ]=\frac{1}{\tau_{\text{TOF}}}+\frac{1}{2\tau_{M,\text{Ndef}}}+\psi \left(\frac{1}{\tau_{Mq}}-\frac{1}{\tau_{M,\text{Ndef}}}\right)$$

The coefficients in Equation (32) can easily be rearranged by using the characteristic times:

(33) $$\tau_{\text{TOF}}=\frac{d^{2}}{\mu_{e}U}$$

(34) $$\tau_{M,\text{Ndef}}=\frac{ɛ{ɛ}_{0}}{\mu_{e}eN_{\text{Def}}}$$

(35) $$\tau_{Mq}=\frac{ɛ{ɛ}_{0}}{\mu_{e}(q_{e}/d)}$$

and their ratios. It can be noticed in Equation (32), that depending on the relations among values of the specific time parameters (Equations (33–35)), and to hold Equation (29) for *q_e_* limitation, three type solutions are obtained as:

(36) $$\psi (t)=\{\begin{array}{c} \psi_{0}\exp[-\frac{t}{\tau_{Mq}}(\frac{\tau_{Mq}}{\tau_{M,\text{Ndef}}}-1)]+\frac{\frac{1}{\tau_{\text{TOF}}}+\frac{1}{2\tau_{M,\text{Ndef}}}}{\frac{1}{\tau_{M,\text{Ndef}}}-\frac{1}{\tau_{Mq}}}\{1-\exp[-\frac{t}{\tau_{Mq}}(\frac{\tau_{Mq}}{\tau_{M,\text{Ndef}}}-1)]\},\text{for}\;\tau_{Mq}>\tau_{M,\text{Ndef}}(A)\\ \begin{array}{l} \psi_{0}+(\frac{1}{\tau_{\text{TOF}}}+\frac{1}{2\tau_{M,\text{Ndef}}})t,\text{for}\;\tau_{Mq}=\tau_{M,\text{Ndef}}(B)\\ \psi_{0}\exp[\frac{t}{\tau_{Mq}}(1-\frac{\tau_{Mq}}{\tau_{M,\text{Ndef}}})]+\frac{\frac{1}{\tau_{\text{TOF}}}+\frac{1}{2\tau_{M,\text{Ndef}}}}{\frac{1}{\tau_{Mq}}-\frac{1}{\tau_{M,\text{Ndef}}}}\{\exp[\frac{t}{\tau_{Mq}}(1-\frac{\tau_{Mq}}{\tau_{M,\text{Ndef}}})]-1\},\text{for}\;\tau_{Mq}<\tau_{M,\text{Ndef}}(C) \end{array} \end{array}$$

These equations (Equations (36)) describe the changes of a dimensionless position 0 ≤ *Ψ* ≤ 1 with time in the interval of 0 ≤ *t* ≤ *t_dr_* for these three (A, B and C) regimes. The corresponding evaluations of a drift time *t_dr_* are obtained by using the relevant boundary condition (*Ψ(t_dr_)* = 1) for Equations (36). These evaluations of a drift time are expressed as follows:

(37) $$t_{dr}=\{\begin{array}{c} \frac{\tau_{Mq}}{\left(\frac{\tau_{Mq}}{\tau_{M,\text{Ndef}}}-1\right)}\ln\frac{\frac{\frac{1}{\tau_{\text{TOF}}}+\frac{1}{2\tau_{M,\text{Ndef}}}}{\frac{1}{\tau_{M,\text{Ndef}}}-\frac{1}{\tau_{Mq}}}-\psi_{0}}{\frac{\frac{1}{\tau_{\text{TOF}}}+\frac{1}{2\tau_{M,\text{Ndef}}}}{\frac{1}{\tau_{M,\text{Ndef}}}-\frac{1}{\tau_{Mq}}}-1},\text{for}\;\tau_{Mq}>\tau_{M,\text{Ndef}}(A)\\ \begin{array}{l} \frac{\left(1-\psi_{0}\right)}{\frac{1}{\tau_{\text{TOF}}}+\frac{1}{2\tau_{M,\text{Ndef}}}},\text{for}\;\tau_{Mq}=\tau_{M,\text{Ndef}}(B)\\ \frac{\tau_{Mq}}{\left(1-\frac{\tau_{Mq}}{\tau_{M,\text{Ndef}}}\right)}\ln\frac{\frac{\frac{1}{\tau_{\text{TOF}}}+\frac{1}{2\tau_{M,\text{Ndef}}}}{\frac{1}{\tau_{Mq}}-\frac{1}{\tau_{M,\text{Ndef}}}}+1}{\frac{\frac{1}{\tau_{\text{TOF}}}+\frac{1}{2\tau_{M,\text{Ndef}}}}{\frac{1}{\tau_{Mq}}-\frac{1}{\tau_{M,\text{Ndef}}}}+\psi_{0}},\text{for}\;\tau_{Mq}<\tau_{M,\text{Ndef}}(C) \end{array} \end{array}$$

These different regimes can be realized by varying the applied voltage *U* (through *τ_TOF_*), the doping *N_Def_* (*τ_M,Ndef_*) and the injected surface charge density *q_e_* (*τ_Mq_*). The first regime A (Equations (36 and 37)) is attributed to a small charge drift. Here, the time dependent variations of the dimensionless position *Ψ(t)* are similar to that of the partially depleted junction. These *Ψ(t)* contain a fast initial increase followed by a saturation character of the *Ψ(t)* changes when *t* approaches to *t_dr_*. The drift time is mainly determined by the injected charge dielectric relaxation time *τ_Mq_*, modified by a mismatch between *τ_Mq_* and *τ_M,Ndef_*. The second regime B can be associated with a correlated drift of the surface charge domain, when *Ψ(t)* increases linearly with *t*, characterized by the invariable *t_dr_*, which is directly determined by *τ_TOF_*. The third regime C is attributed to the large charge drift, when the injected charge is able to locally screen the depletion space charge of ions. Then, *τ_TOF_≅τ_Mq_* < *τ_M,Ndef_*, and the correlated (Ramo's type) drift of the injected rather large charge appears. The large injected charge determines the increasing drift velocity. Then, neither a drift velocity nor acceleration is constant. This leads to an exponentially rising (in time) current density during the charge domain drift time (0 ≤ *t* ≤ *t_dr_*). For this regime, the injected charge density is only limited by values *q_e_* < *q_C_≅C_g_(1 − U_FD_/U)U*. The complete shielding of the external voltage created surface charge *σ* appears if *q_e_* > *q_C_* ≡ *C_g_U*. Only a diffusion of the injected carriers is then possible for *q_e_* > *q_C_*.

The current density ascribed to different regimes can be modelled by using the relevant expressions for *Ψ(t)*, taken from Equations (32) and (36), in the drift velocity equation (Equation (32)). The current density is then expressed as:

(38) $$j_{\text{OFD}}(t)=\{\begin{array}{c} \frac{q_{e}}{\tau_{Mq}}[(\frac{\tau_{Mq}}{\tau_{\text{TOF}}}+\frac{\tau_{Mq}}{2\tau_{M,\text{Ndef}}})+(1-\frac{\tau_{Mq}}{\tau_{M,\text{Ndef}}})\{\psi_{0}\exp[-\frac{t}{\tau_{Mq}}(\frac{\tau_{Mq}}{\tau_{M,\text{Ndef}}}-1)]+\frac{\frac{1}{\tau_{\text{TOF}}}+\frac{1}{2\tau_{M,\text{Ndef}}}}{\left(\frac{1}{\tau_{M,\text{Ndef}}}-\frac{1}{\tau_{Mq}}\right)}\{1-\exp[-\frac{t}{\tau_{Mq}}(\frac{\tau_{Mq}}{\tau_{M,\text{Ndef}}}-1)]\}\}],\text{for}\;\tau_{Mq}>\tau_{M,\text{Ndef}}(A)\\ \begin{array}{l} q_{e}(\frac{1}{\tau_{\text{TOF}}}+\frac{1}{2\tau_{M,\text{Ndef}}}),\;\text{for}\;\tau_{Mq}=\tau_{M,\text{Ndef}};(B)\\ \frac{q_{e}}{\tau_{Mq}}[(\frac{\tau_{Mq}}{\tau_{\text{TOF}}}+\frac{\tau_{Mq}}{2\tau_{M,\text{Ndef}}})+(1-\frac{\tau_{Mq}}{\tau_{M,\text{Ndef}}})\{\psi_{0}\exp[\frac{t}{\tau_{Mq}}(1-\frac{\tau_{Mq}}{\tau_{M,\text{Ndef}}})]+\frac{\frac{1}{\tau_{\text{TOF}}}+\frac{1}{2\tau_{M,\text{Ndef}}}}{\left(\frac{1}{\tau_{Mq}}-\frac{1}{\tau_{M,\text{Ndef}}}\right)}\{\exp[\frac{t}{\tau_{Mq}}(1-\frac{\tau_{Mq}}{\tau_{M,\text{Ndef}}})]-1\}\}],\text{for}\;\tau_{\text{TOF}}≅\tau_{Mq}<\tau_{M,\text{Ndef}}(C) \end{array} \end{array}$$

for the respective regimes A, B and C.

The current density changes within a pulse vertex acquire a relaxation curve shape for the regime A, when screening of electrons (drifting charge domain) by ion charge within the depletion width prevails. For the correlated screening regime B, a square-wave shape current density pulse appears with a flat vertex. While for the correlated (Ramo's type) drift regime C (*τ_TOF_≅τ_Mq_*), the transient with increasing in time current density is inherent.

The monopolar drift of holes can be expressed using methodology described above for the case of electrons drift. The positive charge *q_h_* drift is only possible towards the p^+^-layer. Then, the field for *x* < *X*_0_, which accelerates *q_h_*, is important for the consideration of the induction current. This yields:

(39) $$\sigma =(U-U_{FD})\frac{ɛ{ɛ}_{0}}{d}-q_{h}\psi_{h}$$

The current density, for *U* = const, again acquires the Ramo's-type expression:

(40) $$j=\frac{d\sigma }{dt}=\frac{dC_{\text{Sqh}}}{dt}U=-q_{h}\frac{1}{d}\frac{dX_{h}}{dt}$$

The drift velocity field is described by a differential equation:

(41) $$\frac{d\psi_{h}}{dt}=\frac{\mu_{h}}{d}E_{1}=\frac{\mu_{h}}{d}\left\{-\frac{U}{d}-\frac{1}{2}\frac{eN_{D}d}{ɛ{ɛ}_{0}}+\frac{eN_{D}d}{ɛ{ɛ}_{0}}\psi_{h}+\frac{q_{h}}{ɛ{ɛ}_{0}}(\psi_{h}-1)\right\}=\frac{1}{\tau_{Mq,h}}\left[\psi_{h}\left(\frac{\mu_{h}}{\mu_{e}}\frac{\tau_{Mq,h}}{\tau_{M,\text{Ndef}}}+1\right)-\frac{\tau_{Mq,h}}{\tau_{\text{TOF},h}}-\frac{1}{2}\frac{\mu_{h}}{\mu_{e}}\frac{\tau_{Mq,h}}{\tau_{M,\text{Ndef}}}-1\right]$$

using the characteristic time parameters defined as:

(42) $$\tau_{\text{TOF},h}=\frac{d^{2}}{\mu_{h}U}$$

(43) $$\tau_{Mq,h}=\frac{ɛ{ɛ}_{0}}{\mu_{h}(q_{h}/d)}$$

Assuming the proper boundary conditions:

(44) $$t=0\;\text{for}\;\psi_{h}=\psi_{0}$$

(45) $$t=t_{dr}\;\text{for}\;\psi_{h}=0$$

the solutions of the kinetic equation (Equation (41)) are expressed as follows:

(46) $$\psi_{h}(t)=\psi_{0}\exp\left(\frac{\mu_{h}}{\mu_{e}}\frac{\tau_{Mq,h}}{\tau_{M,\text{Ndef}}}+1\right)\frac{t}{\tau_{Mq,h}}-\left[\exp\left(\frac{\mu_{h}}{\mu_{e}}\frac{\tau_{Mq,h}}{\tau_{M,\text{Ndef}}}+1\right)\frac{t}{\tau_{Mq,h}}-1\right]\left[\frac{\frac{\tau_{Mq,h}}{\tau_{\text{TOF},h}}+\frac{1}{2}\frac{\mu_{h}}{\mu_{e}}\frac{\tau_{Mq,h}}{\tau_{M,\text{Ndef}}}+1}{\frac{\mu_{h}}{\mu_{e}}\frac{\tau_{Mq,h}}{\tau_{M,\text{Ndef}}}+1}\right]$$

The monopolar drift time is then evaluated as:

(47) $$t_{dr,h}=\frac{\tau_{Mq,h}}{\frac{\mu_{h}}{\mu_{e}}\frac{\tau_{Mq,h}}{\tau_{M,\text{Ndef}}}+1}\ln\frac{\frac{\frac{\tau_{Mq,h}}{\tau_{\text{TOF},h}}+\frac{1}{2}\frac{\mu_{h}}{\mu_{e}}\frac{\tau_{Mq,h}}{\tau_{M,\text{Ndef}}}+1}{\frac{\mu_{h}}{\mu_{e}}\frac{\tau_{Mq,h}}{\tau_{M,\text{Ndef}}}+1}}{\frac{\frac{\tau_{Mq,h}}{\tau_{\text{TOF},h}}+\frac{1}{2}\frac{\mu_{h}}{\mu_{e}}\frac{\tau_{Mq,h}}{\tau_{M,\text{Ndef}}}+1}{\frac{\mu_{h}}{\mu_{e}}\frac{\tau_{Mq,h}}{\tau_{M,\text{Ndef}}}+1}-\psi_{0}}$$

The current density of the hole drift is expressed (by inserting Equations (41) and (46) into Equation (30) (modified for holes drift)) as:

(48) $$j_{\text{OFD}}(t)=\frac{q_{h}}{\tau_{Mq,h}}\left[\left\{\psi_{0}\exp\left(\frac{\mu_{h}}{\mu_{e}}\frac{\tau_{Mq,h}}{\tau_{M,\text{Ndef}}}+1\right)\frac{t}{\tau_{Mq,h}}-\left[\exp\left(\frac{\mu_{h}}{\mu_{e}}\frac{\tau_{Mq,h}}{\tau_{M,\text{Ndef}}}+1\right)\frac{t}{\tau_{Mq,h}}-1\right]\left[\frac{\frac{\tau_{Mq,h}}{\tau_{\text{TOF},h}}+\frac{1}{2}\frac{\mu_{h}}{\mu_{e}}\frac{\tau_{Mq,h}}{\tau_{M,\text{Ndef}}}+1}{\frac{\mu_{h}}{\mu_{e}}\frac{\tau_{Mq,h}}{\tau_{M,\text{Ndef}}}+1}\right]\right\}\left(\frac{\mu_{h}}{\mu_{e}}\frac{\tau_{Mq,h}}{\tau_{M,\text{Ndef}}}+1\right)-\frac{\tau_{Mq,h}}{\tau_{\text{TOF},h}}-\frac{1}{2}\frac{\mu_{h}}{\mu_{e}}\frac{\tau_{Mq,h}}{\tau_{M,\text{Ndef}}}-1\right]$$

In the case of the positive charge drift within n-base material, holes are always accelerated due to acting space charge field. The transient is then observed with the current density increasing with time.

#### 2.2.3. Impact of Ion Space Charge in Fully Depleted Detector

The moving charge inside the over depleted space charge layer induces a displacement current component, which exactly compensates the conductivity current component, arisen due to a proximate contacting of the depleted layer with external electrode (outside layer). As can be inferred for the regime B (Equation (38)), characterized by the matched relaxation lifetimes *τ_Mq_* = *τ_M,Ndef_*, the space charge *eN_Def_* over *d* accelerates a drift of the injected charge domain by the shortening of the drift time to the value *t_dr_* = *τ_TOF_/* [1 + (*τ_TOF_/*2*τ_M,Ndef_*)] < *τ_TOF_*, for *Ψ_0_* =0 (Equation (37)).

However, for the regime A of the non-correlated relaxation times of the space charge *eN_Def_* and of the drifting *q_e_/d* one, existence of the space charge *eN_Def_* leads to a reduction of the effective value of the drifting charge:

(49) $$q_{e,ef}=q_{e}\exp(-\frac{t}{\tau_{M,\text{Ndef}}}),\text{for}\;\tau_{Mq}>\tau_{M,\text{Ndef}}$$

Then, a surface density of the drifting charge *q_e,ef_* is instantaneously and locally shielded by the space charge of ions, due to the rapid local reaction of the sufficiently large density charge of ions. Then, an increase ∼ exp[*t*/*τ_Mq_*] of the drift current density (Equation (38)) competes with a seeming reduction of the charge density *q_e_* ∼ exp[−*t*/*τ_M, Ndef_*], caused by the ion space charge. The space charge of ions modifies the current density during *q_e_* drift by varying of length of the *eN_Def_X_e_* bar.

The large injected charge is able to locally screen the depletion space charge of ions, for the regime C. Then, a drift of the injected domain proceeds similarly to that in a capacitor-type device.

#### 2.2.4. Bipolar Drift of Surface Charge in Junction Structure

As usual in detectors, a quasi-neutral domain of the excess carriers is initially generated. Then, owing to a steady-state applied field, these carriers can be separated into the oppositely moving surface charge sub-domains *q_e_* and *q_h_*. These drifting sub-domains induce charges on the electrodes and determine a field in between of them (*q_e_* and *q_h_*), to hold the initial quasi-neutrality. A sketch of the field components is presented in Figure 1a.

An instantaneous electric field distribution along the *x* axis (0 ≤ *x* ≤ d) for the bipolar drift can be represented as:

(50) $$E(x)=\{\begin{array}{c} E_{1}(x)=-\frac{\sigma }{ɛ{ɛ}_{0}}-\frac{eN_{D}}{ɛ{ɛ}_{0}}(d-x)-\frac{q_{h}}{ɛ{ɛ}_{0}},\text{for}\;x<X_{h}\\ E_{3}(x)=-\frac{\sigma }{ɛ{ɛ}_{0}}-\frac{eN_{D}}{ɛ{ɛ}_{0}}(d-x)),\text{for}\;X_{h}<x<X_{e}\\ E_{2}(x)=-\frac{\sigma }{ɛ{ɛ}_{0}}-\frac{eN_{D}}{ɛ{ɛ}_{0}}(d-x)-\frac{q_{e}}{ɛ{ɛ}_{0}},\text{for}\;x>X_{e} \end{array}$$

A field discontinuity at the instantaneous location of surface charge domains is expressed as:

(51) $$E=\{\begin{array}{c} E_{3}(X_{h})-E_{1}(X_{h})=\frac{q_{h}}{ɛ{ɛ}_{0}},\text{for}\;X_{h}\\ E_{2}(X_{e})-E_{3}(X_{e})=-\frac{q_{e}}{ɛ{ɛ}_{0}},\text{for}\;X_{e} \end{array}$$

Then, the relation between the surface charge +*σ* on high potential electrode and the external voltage *U* is obtained by taking the second Poisson integral. The solution for a scalar surface charge density *σ* can be written as:

(52) $$\sigma =(U-U_{FD})\frac{ɛ{ɛ}_{0}}{d}-q_{e}(1-\psi_{e})-q_{h}\psi_{h}\equiv (U-U_{FD})C_{e-h}$$

where the expressions are employed for a full depletion voltage *U_FD_* = *eN_D_d*^2^/2*εε_0_* and for the dimensionless positions *Ψ_e,h_* = *X_e,h_/d* of the sub-domains. However, the full depletion voltage value may become variable in the case of carrier capture/emission due to a change of effective dopant density *N_D_* = *N_Def_*.

It is worth to point out, that in the case of the bipolar drift, the charge *σ* on the high potential electrode becomes dependent on the instantaneous location of both electron and hole separated domains *σ(Ψ_e_, Ψ_h_)*. This leads to the appearance of the fields acting on electrons (*E_2_*) and holes (*E_1_*). Theses fields also depend on the instantaneous location of the drift counter-partners as:

(53) $$E(\psi )=\{\begin{array}{c} E_{1}=-\frac{U+U_{FD}}{d}+\frac{q_{e}}{ɛ{ɛ}_{0}}(1-\psi_{e})-\frac{q_{h}}{ɛ{ɛ}_{0}}(1-\psi_{h})+\frac{eN_{D}d}{ɛ{ɛ}_{0}}\psi ,\text{for}\;x<X_{h}\\ E_{2}=-\frac{U+U_{FD}}{d}-\frac{q_{e}}{ɛ{ɛ}_{0}}\psi_{e}+\frac{q_{h}}{ɛ{ɛ}_{0}}\psi_{h}+\frac{eN_{D}d}{ɛ{ɛ}_{0}}\psi ,\text{for}\;x>X_{e} \end{array}$$

The induced charge current density, due to a bipolar drift, is expressed as follows:

(54) $$j=\frac{d\sigma }{dt}=-(q_{h}\frac{1}{d}\frac{dX_{h}}{dt}-q_{e}\frac{1}{d}\frac{dX_{e}}{dt})$$

It can be noticed that, owing to v_e_ = −v_h_, the scalar current density can be represented by a sum of Ramo's-type components:

(55) $$j=q(\frac{d\psi_{h}}{dt}+\frac{d\psi_{e}}{dt})$$

The bipolar drift velocities are correlated during the bipolar drift time *τ_b_* (within time *τ_b_* domain) as:

(56) $$\frac{\left(1-\psi_{e}\right)}{\frac{d\psi_{e}}{dt}}=\frac{\psi_{h}}{-\frac{d\psi_{h}}{dt}}=\tau_{b}$$

Here, the drift directions are included by accepting the relevant sign for the scalar velocity. There exist several situations of the pure bipolar and the mixed drift regimes. These regimes can be separated as:

(57) $$\frac{\psi_{0}}{-\frac{d\psi_{h}}{dt}}=\frac{\left(1-\psi_{0}\right)}{\frac{d\psi_{e}}{dt}}=\tau_{b},\;\text{for}\;\tau_{b}=\tau_{dr,e}=\tau_{dr,h}$$

(58) $$\frac{\psi_{0}}{-\frac{d\psi_{h}}{dt}}=\frac{\left(1-\psi_{e}\right)}{\frac{d\psi_{e}}{dt}}=\tau_{dr,h},\;\text{for}\;\tau_{b}=\tau_{dr,h}<\tau_{dr,e}$$

(59) $$\frac{\psi_{h}}{-\frac{d\psi_{h}}{dt}}=\frac{\left(1-\psi_{0}\right)}{\frac{d\psi_{e}}{dt}}=\tau_{dr,e},\;\text{for}\;\tau_{b}=\tau_{dr,e}<\tau_{dr,h}$$

The regime (Equation (57)) of the synchronous drift of both type carriers within the entire inter-electrode gap can only be realized for a single definite point of the charge domain injection *Ψ_0_*. While the mixed drift processes appear when the bipolar drift (within current pulse) changes to either the monopolar drift of the electron domain after holes reach a p^+^-layer or it becomes the monopolar drift of the hole domain after electrons reach the high potential electrode. These latter situations depend on the injection location *Ψ_0_* = *Ψ_0h_* = *Ψ_0e_* within n-base region of the junction and on the mobility of carriers. In the general case of the junction structure, the drift process in both layers of the junction should be included. For instance, a drift of holes within n-base region should be extended into p^+^-layer of the abrupt junction to exactly account for the bipolar and monopolar regimes.

##### Pure Bipolar Drift

In the case of the pure bipolar drift regime (Equation (57)), a system of kinetic equations for the n-base region and their solutions are expressed as follows:

(60) dψhdt=−ψ0τb;ψh=ψ(1−tτb)0

(61) $$\frac{d\psi_{e}}{dt}=\frac{1-\psi_{0}}{\tau_{b}};\psi_{e}=\psi_{0}(1-\frac{t}{\tau_{b}})+\frac{t}{\tau_{b}}$$

These solutions should satisfy the boundary conditions:

(62) ψh|t=0=ψ;0ψh|t=τb=0

(63) $$\psi_{e}{|}_{t=0}=\psi_{0};\psi_{e}{|}_{t=\tau_{b}}=1$$

However, in the more precise approximation, the monopolar drift of holes within p^+^-region included by *Ψ_0_* = *Ψ_0n_* + *Ψ_0p_*_+_, should be analyzed. Therefore, in rigorous consideration, the pure bipolar drift can be assumed as an idealization. Nevertheless, for the case of *τ_tr,hp_*_+_ ≪ *τ_tr,hn_*, the single layer approximation can be a relevant approach.

Then, the inherent time *τ_b_* of the bipolar drift is obtained by integrating an expression for the drift velocity (using Equations (55, 57, 60 and 61)) as:

(64) dψhdt=μhdE1∫ψ00dψh=∫0τbμhd[−Ud−12eNDdɛɛ0+qeɛɛ0(1−[ψ0(1−tτb)+tτb])−qhɛɛ0(1−[ψ(1−tτb)0])+eNDdɛɛ0ψ0(1−tτb)]dtτb=ψ0τTOF,h1+12μhμeτTOF,h(1τM,Ndef−1τMq,e)(1−ψ0)+τTOF,hτMq,h(1−ψ0)+12τTOF,hτMq,hψ0≈ψ0τTOF,h

Inserting these solutions (Equations (60, 61 and 64)) into Equation (55), the current density (in the case of the pure bipolar drift) is expressed as:

(65) $$j=\frac{q}{\tau_{b}}=\frac{q}{\psi_{0}\tau_{\text{TOF},h}}\{1+\frac{1}{2}\frac{\mu_{h}}{\mu_{e}}\tau_{\text{TOF},h}(\frac{1}{\tau_{M,\text{Ndef}}}-\frac{1}{\tau_{Mq,e}})(1-\psi_{0})+\frac{\tau_{\text{TOF},h}}{\tau_{Mq,h}}(1-\psi_{0})+\frac{1}{2}\frac{\tau_{\text{TOF},h}}{\tau_{Mq,h}}\psi_{0}\}\approx \frac{q}{\psi_{0}\tau_{\text{TOF},h}}$$

Thus, the pure bipolar drift leads to an invariable current density with pulse duration of *t_P_≅Ψ_0_τ_TOF,h_*, Equation (65), provided that carrier capture can be ignored. The dimensionless position of the charge injection always is *Ψ_0_* ≤ 1. Therefore, the injected charge drift current pulse is even shorter than a time of flight of the counter-partners in bipolar drift, *i.e.*, either *t_P_* < *τ_TOF,h_* or *τ_TOF,e_*(1−*Ψ_0_)*.

##### Bipolar Drift During Hole Drift Time

In the case of the hole drift time *τ_tr,h_* is the shortest one among the characteristic times, a bipolar (*τ_bB_* =*τ_tr,h_*) drift (Equation (58)) is described by a system of the kinetic equations and their solutions, which can be presented as follows:

(66) dψhdt=−ψ0τtr,h;ψh=ψ(1−tτb)0

(67) $$\frac{d\psi_{e}}{dt}=\frac{1-\psi_{e}}{\tau_{tr,h}};\psi_{e}=\psi_{0}\exp(-\frac{t}{\tau_{tr,h}})+[1-\exp(-\frac{t}{\tau_{tr,h}})]$$

These solutions should satisfy the boundary conditions:

(68) ψh|t=0=ψ;0ψh|t=τtr,h=0

(69) $$\psi_{e}{|}_{t=0}=\psi_{0};\psi_{e}{|}_{t=\tau_{tr,h}}\equiv \psi_{e}^{*0}{(}_{t=\tau_{tr,h}})=[1-\exp(-1)]+\psi_{0}\exp(-1)$$

Here, Ψ_e_*^0^ serves as the start position for a drift of the electron domain, just during an instant of disappearing of the domain of holes at the grounded electrode. The time *τ_bB_* of the initial bipolar drift is obtained by integrating the expression for the drift velocity (using Equations (66–69 and 55)), as:

(70) $$\begin{array}{l} \frac{d\psi_{h}}{dt}=\frac{\mu_{h}}{d}E_{1}\\ \int_{\psi_{0}}^{0}dy_{h}=\int_{0}^{\tau_{tr,h}}\{\frac{\mu_{h}}{d}[-\frac{U}{d}-\frac{1}{2}\frac{eN_{D}d}{ɛ{ɛ}_{0}}+\psi_{0}(1-\frac{t}{\tau_{b}})(\frac{eN_{D}d}{ɛ{ɛ}_{0}}+\frac{q_{h}}{ɛ{ɛ}_{0}})-\frac{q_{e}}{ɛ{ɛ}_{0}}(\psi_{0}\exp(-\frac{t}{\tau_{tr,h}})+(1-\exp(-\frac{t}{\tau_{tr,h}}))]\}dt\\ \tau_{bB}\equiv \tau_{tr,h}=\frac{\psi_{0}\tau_{\text{TOF},h}}{1+\frac{\mu_{h}}{\mu_{e}}\tau_{\text{TOF},h}\left[\frac{1}{2\tau_{M,\text{Ndef}}}-\frac{\psi_{0}}{2\tau_{M,\text{Ndef}}}+\frac{1}{\tau_{Mq,e}}+\frac{1}{\tau_{Mq,e}}(1-\psi_{0})(e^{-1}-1)\right]-\frac{\psi_{0}}{2}\frac{\tau_{\text{TOF},h}}{\tau_{Mq,h}}} \end{array}$$

Here, the step-like change of field and current density would have obtained for an instant of hole arrival to the grounded electrode. To validate the charge, charge momentum, and energy conservation, the coordinate transform should be performed, to stitch the solutions obtained in the moving (*τ_bB_* time domain) coordinate system to that obtained (Equation (30)) for the system of coordinates at rest. This transform should include the charge induction on the electrodes, the drift velocity conservation and the coordinate relations, which can be represented as:

(71) $$\sigma^{+}=\sigma +q$$

(72) $${\frac{d\psi^{+}}{dt}=(\frac{d\psi_{h}}{dt}+\frac{d\psi_{e}}{dt})|}_{\tau b}$$

(73) $$\psi^{+}=-\frac{1}{1-\frac{\tau_{Mq,e}}{\tau_{M,\text{Ndef}}}}+(\psi_{e}-\psi_{e_{e}}^{*0}),\tau_{Mq,e}<\tau_{M,\text{Ndef}}$$

(74) $$-\psi^{+}=-\frac{1}{\frac{\tau_{Mq,e}}{\tau_{M,\text{Ndef}}}-1}-(\psi_{e}-\psi_{e_{e}}^{*0}),\tau_{Mq,e}>\tau_{M,\text{Ndef}}$$

These transforms relate the “new” *Ψ*^+^ coordinate in the system at rest with that *Ψ* (within bipolar drift time domain) moving one for the proceeded monopolar drift analysis, after the charge induction procedure is accounted for, and velocities are matched. Then the prolonged monopolar drift velocity of electrons (for the case when *τ_Mq,e_* < *τ_M,Ndef_*) is expressed as:

(75) $$\begin{array}{ll} \frac{d\psi_{e,\text{mon}}}{dt} & =\frac{v_{0,e,\text{mon}}}{d}+\frac{1}{\tau_{Mq,e}}[\frac{\tau_{Mq,e}}{\tau_{\text{TOF},e}}+\frac{1}{2}\frac{\tau_{Mq,e}}{\tau_{M,\text{Ndef}}}-1+(\psi_{e,\text{mon}}-\psi_{e}^{*0})(1-\frac{\tau_{Mq,e}}{\tau_{M,\text{Ndef}}})]=\\ & =\frac{v_{0,e,\text{mon}}}{d}+\frac{1}{\tau_{Mq,e}}[R_{e1}+\frac{1}{2}R_{e2}-1+(\psi_{e,\text{mon}}-\psi_{e}^{*0})(1-\frac{\tau_{Mq,e}}{\tau_{M,\text{Ndef}}})] \end{array}$$

Here, *R_e1_* and *R_e2_* are the lifetime ratios, which can be dependent on the voltage drop sharing; *v_0,e,mon_* is the initial velocity of the monopolar electron drift, which is obtained using relations of velocity vectors and their directions for bipolar drift and for the re-calibrated monopolar drift as:

(76) $${{\vec{v}}_{e,\text{mon}}|}_{t=\tau_{b,B}}={\vec{v}}_{e,\text{bip}}-{\vec{v}}_{h,\text{bip}}$$

(77) $$\left|{\vec{v}}_{0,e,\text{mon}|\psi_{e}^{*0}}\right|=\left|{\vec{v}}_{e,\text{bip},|\psi_{e}^{*0},\tau_{b}}\right|+\left|{\vec{v}}_{h,\text{bip}|0,\tau_{b}}\right|=v_{\Sigma \text{bip}}{|}_{\psi_{e}^{*0}}$$

This gives a coincidence of *v_0,e,mon_* and *v_Σbip_*|*_Ψ_*_e_*^0^ values at the position Ψ_e_*^0^ of an electron domain. Expressions for the function *ψ(t)* and monopolar drift time are obtained by integrating (Equation (75)) expression with the initial and boundary conditions:

(78) $$\psi_{e}{|}_{t=0}=\psi_{e}^{*0};\psi_{e}{|}_{t=\tau e,\text{mon}}=1$$

These solutions are given as:

(79) $$\psi_{e,\text{mon}}=(\exp((1-\frac{\tau_{Mq,e}}{\tau_{M,\text{Ndef}}})\frac{t}{\tau_{Mq,e}})-1)(\frac{\frac{\tau_{Mq,e}}{\tau_{\text{TOF},e}}+\frac{1}{2}\frac{\tau_{Mq,e}}{\tau_{M,\text{Ndef}}}-1+\frac{v_{0}\tau_{Mq,e}}{d}}{1-\frac{\tau_{Mq,e}}{\tau_{M,\text{Ndef}}}})+\psi_{e}^{*0}$$

(80) $$\tau_{e,\text{mon}}=\frac{\tau_{Mq,e}}{1-\frac{\tau_{Mq,e}}{\tau_{M,\text{Ndef}}}}\ln(\frac{\frac{\frac{\tau_{Mq,e}}{\tau_{\text{TOF},e}}+\frac{1}{2}\frac{\tau_{Mq,e}}{\tau_{M,\text{Ndef}}}-1+\frac{v_{0}\tau_{Mq,e}}{d}}{\left(1-\frac{\tau_{Mq,e}}{\tau_{M,\text{Ndef}}}\right)}+1-\psi_{e}^{*0}}{\frac{\frac{\tau_{Mq,e}}{\tau_{\text{TOF},e}}+\frac{1}{2}\frac{\tau_{Mq,e}}{\tau_{M,\text{Ndef}}}-1+\frac{v_{0}\tau_{Mq,e}}{d}}{\left(1-\frac{\tau_{Mq,e}}{\tau_{M,\text{Ndef}}}\right)}})$$

The entire duration *t_P_* of the current pulse contains the both phases:

(81) $$t_{P}=\tau_{bB}+\tau_{e,\text{mon}}$$

of the bipolar (*τ_bB_*) and the monopolar (*τ_e_*,_mon_) drift of electrons. Inserting these solutions (Equations (66–70)) into Equation (55) for the monopolar drift of electrons with Ψ_e_*^0^ as an initial position, the current density (in the case of the mixed regime (Equation 58)) is expressed as:

(82) $$j(t)=\{\begin{array}{c} \begin{array}{l} j_{1}=\frac{q_{e}}{\tau_{tr,h}}\{\psi_{0}[1-\exp(-\frac{t}{\tau_{tr,h}})]+\exp(-\frac{t}{\tau_{tr,h}})\},\text{for}0\leq t\leq \tau_{tr,h} \end{array}\\ j_{2}=\frac{q_{e}}{\tau_{Mq,e}}[\exp(1-\frac{\tau_{Mq,e}}{\tau_{M,\text{Ndef}}})\frac{t}{\tau_{Mq,e}}(\frac{\tau_{Mq,e}}{\tau_{\text{TOF},e}}+\frac{1}{2}\frac{\tau_{Mq,e}}{\tau_{M,\text{Ndef}}}-1+\frac{v_{0}\tau_{Mq,e}}{d})],\text{for}0\leq t\leq \tau_{e,\text{mon}}. \end{array}$$

The current transient (for the analyzed case of *τ_Mq,e_* < *τ_M,Ndef_*) has a decreasing current component within the initial phase (during a bipolar drift) and an increasing one within the rearward component of the transient due to a drift of the electron domain which screens the space charge. For the case when *τ_Mq,e_* = *τ_M,Ndef_*, the electrons drift with the constant initial velocity *v_0,e,mon_* due to a compensation of the drifting charge and the space charge fields. For the case of *τ_Mq,e_* > *τ_M,Ndef_*, the electrons drift with a decreasing velocity, as the large space charge of ions (relative to a drifting charge) screens the drifting charge.

##### Bipolar Drift During Drift Time of Electrons

In the case the electron drift time *τ_tr,e_* is the shortest one among the characteristic times, a bipolar (*τ_bC_* = *τ_tr,e_*) drift is followed by a monopolar drift of holes towards p^+^ layer of the junction. After performing analogous (as described in previous section) coordinate transformations and solving drift velocity equations for the bipolar (during drift time of electrons (Equation 59)) and prolonged monopolar drift of holes, the current density is expressed as:

(83) $$j=\{\begin{array}{c} j_{1}=\frac{q_{h}}{\tau_{tr,e}}\{1-\psi_{0,h}[1-\exp(-\frac{t}{\tau_{tr,e}})]\},\text{for}0\leq t\leq \tau_{tr,e}\\ \begin{array}{l} j_{2}=\frac{q_{h}}{\tau_{Mq,h}}[\exp((\frac{\mu_{h}}{\mu_{e}}\frac{\tau_{Mq,h}}{\tau_{M,\text{Ndef}}}+1)\frac{t}{\tau_{Mq,h}})\{-\frac{\tau_{Mq,h}}{\tau_{\text{TOF},h}}-\frac{1}{2}\frac{\mu_{h}}{\mu_{e}}\frac{\tau_{Mq,h}}{\tau_{M,\text{Ndef}}}+(\frac{\mu_{h}}{\mu_{e}}\frac{\tau_{Mq,h}}{\tau_{M,\text{Ndef}}}+1)-(\frac{\mu_{h}}{\mu_{e}}\frac{\tau_{Mq,h}}{\tau_{M,\text{Ndef}}}+1)\psi_{h}^{*0}+\frac{v_{0}\tau_{Mq,h}}{d}\}\\ +\psi_{h}^{*0}(\frac{\mu_{h}}{\mu_{e}}\frac{\tau_{Mq,h}}{\tau_{M,\text{Ndef}}}+1)\exp((\frac{\mu_{h}}{\mu_{e}}\frac{\tau_{Mq,h}}{\tau_{M,\text{Ndef}}}+1)\frac{t}{\tau_{Mq,h}})],\text{for}0\leq t\leq \tau_{h,\text{mon}} \end{array} \end{array}+$$

with the entire duration *t_P_* of the current pulse:

(84) $$t_{P}=\tau_{bC}+\tau_{h,\text{mon}}$$

which consists of the components of the bipolar drift time:

(85) $$\tau_{b,C}\equiv \tau_{tr,e}=\frac{(1-\psi_{0})\tau_{\text{TOF},e}}{1+\frac{\tau_{\text{TOF},e}}{2\tau_{M,\text{Ndef}}}+(\frac{1}{\tau_{Mq,e}}-\frac{1}{\tau_{M,\text{Ndef}}})\tau_{\text{TOF},e}\psi_{0}+(1-\psi_{0})\tau_{\text{TOF},e}(\frac{1}{\tau_{Mq,e}}-\frac{1}{\tau_{M,\text{Ndef}}})\frac{1}{2}-\frac{\mu_{e}}{\mu_{h}}\frac{\tau_{\text{TOF},e}}{\tau_{Mq,e}}(1-e^{-1})}$$

and the monopolar drift time:

(86) $$\tau_{e,\text{mon}}=\frac{\tau_{Mq,h}}{\frac{\mu_{h}}{\mu_{e}}\frac{\tau_{Mq,e}}{\tau_{M,\text{Ndef}}}+1}\ln(\frac{\frac{-\frac{\tau_{Mq,h}}{\tau_{\text{TOF},h}}-\frac{1}{2}\frac{\mu_{h}}{\mu_{e}}\frac{\tau_{Mq,h}}{\tau_{M,\text{Ndef}}}+(\frac{\mu_{h}}{\mu_{e}}\frac{\tau_{Mq,h}}{\tau_{M,\text{Ndef}}}+1)-(\frac{\mu_{h}}{\mu_{e}}\frac{\tau_{Mq,h}}{\tau_{M,\text{Ndef}}}+1)\psi_{h}^{*0}+\frac{v_{0}\tau_{Mq,h}}{d}}{\left(\frac{\mu_{h}}{\mu_{e}}\frac{\tau_{Mq,h}}{\tau_{M,\text{Ndef}}}+1\right)}}{\frac{-\frac{\tau_{Mq,h}}{\tau_{\text{TOF},h}}-\frac{1}{2}\frac{\mu_{h}}{\mu_{e}}\frac{\tau_{Mq,h}}{\tau_{M,\text{Ndef}}}+(\frac{\mu_{h}}{\mu_{e}}\frac{\tau_{Mq,h}}{\tau_{M,\text{Ndef}}}+1)-(\frac{\mu_{h}}{\mu_{e}}\frac{\tau_{Mq,h}}{\tau_{M,\text{Ndef}}}+1)\psi_{h}^{*0}+\frac{v_{0}\tau_{Mq,h}}{d}}{\left(\frac{\mu_{h}}{\mu_{e}}\frac{\tau_{Mq,h}}{\tau_{M,\text{Ndef}}}+1\right)}+\psi_{h}^{*0}})$$

Here, Ψ_h_*^0^ denotes the initial domain position within the monopolar drift of holes; *v_0,h,mon_* is the initial velocity of the monopolar hole drift which is obtained using the relations of velocity vectors and their directions for the bipolar drift and for the re-calibrated monopolar drift as:

(87) $$\left|{\vec{v}}_{0,h,\text{mon}|\psi_{h}^{*0}}\right|=\left|{\vec{v}}_{e,\text{bip},\tau_{b}}\right|+\left|{\vec{v}}_{h,\text{bip},\tau_{b}}{|}_{\psi_{h}^{*0}}\right|=v_{\Sigma \text{bip}}$$

In the case of the proceeded hole monopolar drift within n-base material (after the phase of the bipolar drift is finished), holes are always accelerated due to the acting space charge field. The hole drift with an increasing velocity determines an inherent shape of the increasing current density within a transient, during the monopolar drift phase.

## 3. The Impact of Carrier Trapping and Generation

### 3.1. The Impact of Carrier Trapping

The injected charge current can also be changed by carrier trapping and generation. The surface charge density dependence on time for the simple traps can be expressed as:

(88) $$q_{e}(t)=q_{e0}\exp(-t/\tau_{C}).$$

Introducing a trapping dependent dielectric relaxation time as:

(89) $$\tau_{Mq,tr}=\frac{ɛ{ɛ}_{0}}{\mu_{e}(q_{e0}/d)\exp(-t/\tau_{C})}=\tau_{Mq}\exp(t/\tau_{C})$$

the time dependent changes of the charge on the high potential electrode are described as:

(90) $$\sigma (t)=U\frac{d}{ɛ{ɛ}_{0}}-\frac{eN_{\text{Def}}(t)d}{2}-q_{e}(t)(1-\psi_{e}(t))$$

Here, the time dependent quantities of *N_Def_(t)* and *q_e_(t)* should be employed. Capture of the injected excess carriers, as usual, leads to a synchronous filling of empty donor and acceptor type traps those determine the overall charge neutrality and effective doping *N_Def_*. Then, the current density within a pulse, at assumption that *N_Def_(t)*= *N_Def0_*exp(−*t/τ_C_*), is expressed as:

(91) jOFD,tr(t)=dσdt=qe(t)τC(1−X0(t)d)+eN(t)Defd2τC+qe(t)1ddX0(t)dt

This equation should be properly matched with the drift kinetic equation:

(92) $$\frac{d\psi }{dt}-\psi \left(\frac{\exp(-t/\tau_{C})}{\tau_{Mq}}-\frac{\exp(-t/\tau_{C})}{\tau_{M,\text{Ndef}}}\right)-\frac{1}{\tau_{\text{TOF}}}-\frac{\exp(-t/\tau_{C})}{2\tau_{M,\text{Ndef}}}=0$$

The latter equation can only be solved numerically, although the general solution [17] can be expressed through complicated integrals [16].

A few aspects of the impact of carrier trapping on the injected charge transients for a partially depleted junction layer have been mentioned in [14,18]. Evaluation of other parameters (e.g., *t_dr_*) of the transients determined by the injected charge drift and trapping becomes even more complicated, and it can be implemented only by the numerical methods. For prevailing of trapping processes, no articulated features of the detector response ascribed to the injected domain drift can be separated, and only the relaxation-type shape followed the charge domain injection peak can be observable within a current transient.

No drift exists (*∂Ψ/∂t* ≅ 0) and, consequently, Ramo's current component disappears, if a trapping lifetime of the induced charge *q_e0_* domain is the shortest one within a set of characteristic relaxation parameters. Then, equation (Equation (91)) for current density can be simplified as:

(93) $$j_{tr}(t)=\frac{q_{e0}\exp(-t/\tau_{C})}{\tau_{C}}$$

which describes the induction current, - due to the local changes of the injected *q_e_(t)* charge density.

### 3.2. Generation Current Component

Carrier trapping, associated with a drifting surface charge domain, may determine the immediate (during time significantly shorter than other characteristic time parameters) and local changes of the effective charge. A decrease of *N_Def_* due to a filling of the charged donor-type traps is equivalent to a local charge generation. Thus a simplified approach for evaluation of the generation current can be considered. Then, carrier trapping and generation can be analyzed synchronously by rearranging Equation (88) as:

(94) $$q_{e}(t)=q_{e0}[\exp(-t/\tau_{C})+em_{0}d\exp(t/\tau_{g,ef})]$$

Here, *m_0_* denotes the initial carrier density on filled traps or the density of the neutralized donor-type traps, *e* is the elementary charge, and *τ_g,ef_* is an effective generation lifetime. At these simplified assumptions, Equation (94) can further be rearranged as:

(95) $$q_{e}(t)=q_{e0}\exp(-t/\tau_{C})[1+g_{m0}\exp(t/\tau_{gC,ef})]\equiv q_{e0}\exp(-t/\tau_{C})f(t)$$

with the additional designations as:

(96) $$g_{m0}=\frac{em_{0}d}{q_{e0}};$$

(97) $$\tau_{gC,ef}={\left(\frac{1}{\tau_{C}}+\frac{1}{\tau_{g,ef}}\right)}^{-1};$$

(98) $$f(t)=[1+g_{m0}\exp(t/\tau_{gC,ef})].$$

This simplified approach enables one to include into consideration the local charge generation. Unfortunately, the solutions can be obtained only by numerical analysis.

The discussed above simplified analysis of the components of carrier drift, trapping and thermal release enables one to make the rough estimations of the impact of different components. However, the rigorous consideration of processes should be based on the causality principle. The current changes can only appear during or after injection of *q_e_*. Therefore all the relations for the electric fields and charges caused by *q_e_*, obtained within the electrostatic approach, and containing the time dependent *q_e_(t*) components should appear as the convolution integrals, for instance, as:

(99) j(t)=dσdt=1τL∫0tqe(Θ)τC(1−ψ(t−Θ))dΘ+eN(t)Defd2τC−1τL∫0tqe(Θ)dψ(t−Θ)dtdΘ(99)

(100) $$\frac{d\psi }{dt}-\frac{1}{\tau_{L}}\int_{0}^{t}\frac{\exp[-(t-\Theta )/\tau_{C}]}{\tau_{Mq}(\Theta )}\psi (t-\Theta )d\Theta -\frac{1}{\tau_{L}}\int_{0}^{t}\frac{\exp[-(t-\Theta )/\tau_{C}]}{\tau_{M,\text{Ndef}}(t-\Theta )}d\Theta -\frac{1}{\tau_{\text{TOF}}}=0$$

This specification leads to the integral-differential equations. These equations can only be analyzed numerically. Then, the above presented simplified models can be employed for the initial and qualitative prediction of the numerical solutions.

## 4. The Role of Carrier Diffusion

The drift velocity is varied through the electrostatic interaction of injected charge *q_e_* and surface charge *σ* on electrode, due to external voltage source. The initial zero drift velocity actually appears when the detector signals are caused by the secondary electron-hole pairs generated by the energetic elementary particles (high energy photons, hadrons, *etc.*). Then, a neutral domain with an equal density of electrons and holes in pairs is locally generated. The external field is able to separate and move these counter-partners towards opposite directions if the density of these carriers is less than *q_C_*, *i.e.*, for *q_e_* < *q_C_* = *C_g_U*.

In the partially depleted junction layer, for *U* ≤ *U_FD_*, the current (ascribed to the injected charge drift) varies due to the temporal changes in *w_q_(t)*, and it really contains a pair separation *X_e-h_(t)* = *X_e_−X_h_* length. The charge separation process induces the change of the depletion width *w_q_* (an increase, relatively to its steady-state value *w_0,n&p_*_+_) in both layers of the junction, *i.e.*, *w_q_,_n_* and *w_q_,_p_*_+_. The extracted excess holes are located at p^+^-side producing the same value of the surface field. Thus, the overall charge balance *w_p_* + *N^-^_A,p_*_+_ = *w_n_N*^+^*_D,n_* together with *q_h,p_* + *X_0,p_*_+_ = *q_e,n_X_0,n_* is maintained in a diode for the moderate density of the injected charge domain. In the heavily doped layer, the characteristic times, *e.g.*, dielectric relaxation times, are significantly shorter than those in the resistive layer. Therefore, current transient pulse duration is mainly determined by the longer processes within more resistive layer. This motivates an approach of a separate consideration of electron drift within a base region of the reverse biased diode detector, where a charge separation process is assumed to be sufficiently short, and a drift starts after extraction of counter-partners (separation of pairs) process is finished.

In the general case of the local injection of excess carrier pairs, separation of counter-partners depends on their densities. The external source induced charge *σ* on electrode can be completely shielded by the large injected charge *q_e_* within a Debye screening length during the dielectric relaxation on metallic electrode, which is extremely short. The space charge of ions is also screened during *τ_Mq_*, which is then the shortest one among the characteristic times, in the case of *q_e_* ≫ *eN_Def_d* and *(q_e_/ε_0_ε)d* > *U*. The external source is able to react by changing *σ*, till the system capacitance is *C_Sq_* > 0. However, the large injected charge *q_e_* nearby the grounded electrode (*X_0_* ≅ 0) is able to create an internal field and a voltage drop *(q_e_/ε_0_ε)d*, which reduces the dynamic capacitance of the system to *C_Sq_* = 0. Then, the external voltage source is completely blocked in supporting of *σ*. As a result, no separation of the electron-hole cloud (into domains of electrons and holes) appears. Thereby, relaxation of the injected quasi-neutral domain happens completely by carrier diffusion process.

The reason is the excess carrier diffusion and appearance of the diffusion induced inner field [19] as:

(101) $${\vec{E}}_{D}=\frac{1}{p\mu_{h}+n\mu_{e}}(D_{h}\vec{\nabla }p-D_{e}\vec{\nabla }n)$$

This field is proportional to the excess carrier density gradients. For the locally generated domain of the nearly infinitesimal width (for instance in tracking of hadron path) within significantly wider inter-electrode gap of detector, diffusion due to a sharp gradient (which is also proportional to the carrier density) induces an inner electric field which balances a further widening of the domain. Strength of this field can be sufficient to compensate partially or fully the applied external field (surface charge *σ*) in the case of low applied voltage and the rather high densities of the injected carriers. Then, carrier diffusion and drift leads to the outspread of the domain. Such a process is characterized by the ambipolar diffusion coefficient *D_a_*. In thin detectors with small applied dc voltage, a width of the outspread domain can approach the geometrical dimensions of the inter-electrode layer, even when it is formed by the strongly absorbed radiation. The same situation can be realized by the photo-injection of excess carrier into a rather thick detector using the homogenously absorbed light. Then, the injected domain sweeps the inter-electrode spacing. The external electric field acts as the accelerating factor for the surface recombination *sd/D* (of velocity *s*, related to *sd/D* = *d/L_D_* by Debye screening length *L_D_*). This problem is very similar to the excess carrier ambipolar diffusion moderated carrier recombination on surfaces. Then, action of the external field and carrier drift can be included into the properly modified surface recombination velocity *s*. Solution of this problem is well-known [20–23], and it leads to a transient of conductivity current due to the carriers arrived to the electrode and extracted to its surface. Time variations of the excess carrier density in this domain (averaged over the geometrical thickness *d* of inter-electrode space) is expressed through a sum of the space mode *η_m_* components as:

(102) $$n_{ex}(t)=n_{ex}(t=0)\sum_{m=1}^{\infty }A_{m}\exp(-\eta_{m}^{2}D_{a}t)$$

The space frequencies (*η*) of these decay modes are described by the solution of the transcendental equations of type:

(103) $$\text{ctg}\;\eta d=\frac{s_{1}}{s_{1}+s_{2}}(\frac{D\eta }{s_{1}}-\frac{s_{2}}{D\eta })$$

Here, *s_1_* = *L_D,h_/τ_TOF,LD,h_* and *s_2_* = *L_D,e_/τ_TOF,LD,e_* denote the surface recombination velocities ascribed to the electric field caused extraction of carriers towards the front and rear (surfaces) electrodes (with relevant Debye lengths *L_D,h_, L_D,e_*), respectively. These (Equation (102)) solutions with roots found from Equation (103) predict a two-componential, the relaxation type current transient. Such a transient contains the initial non-exponential relaxation component. The asymptotic decay component is characterized by the time parameter *τ_D_* = 1*/η_1_^2^D* of the main decay mode, representing the effective time of the domain dissipation,— due to diffusion over the entire inter-electrode spacing.

## 5. Current Transient Changes Determined by a Signal Recording Circuit

A signal registration circuit (namely, load resistor) inevitably transforms the current transient shape. This appears due to the voltage sharing and the consequent change of a voltage drop on detector depending on current value within the circuit. In more general case, the transients are described by the solutions of the differential equation with variable coefficients, derived as:

(104) $$\frac{\sigma (t)}{C_{Sq}(t)}+iR_{L}=U,\text{withi}=S\frac{d\sigma }{dt}$$

(105) $$\frac{1}{C_{Sq}}\frac{d\sigma (t)}{dt}-\frac{\sigma (t)}{C_{Sq}}\frac{dC_{Sq}(t)}{C_{Sq}dt}+\frac{di}{dt}R_{L}=0,\text{with}U_{CR}=U-iR_{L},U_{CR}=\frac{\sigma (t)}{C_{Sq}}$$

This leads to a differential equation:

(106) $$\frac{di}{dt}+(\frac{d\ln SC_{Sq}(t)}{dt}+\frac{1}{R_{L}SC_{Sq}(t)})i=\frac{U}{R_{L}}\frac{d\ln SC_{Sq}(t)}{dt}$$

which should be solved by using the initial conditions:

(107) $$i_{0}(t=0)=0$$

(108) $$SC_{Sq}(t=0,q_{e}=0)=C_{0}$$

for the ascending component of a transient, and:

(109) $$i_{0,r}(t=t_{dr})=j(t=t_{dr})S=i(t_{dr})$$

(110) $$SC_{Sq,r}(t=t_{dr},\psi_{e}=1)=C_{0}$$

for the relaxation stage of the transient, respectively.

The changes of a system dynamic capacitance determine the initial delay and the final stage (relaxation) components within the simulated transient. These components are inevitable within the charge drift current transients, recorded in experiments. Also, these components should be included into the evaluation of the charge collection efficiency. Depending on the geometrical capacitance (*C_g_*) and load resistance (*R*_L_) values, the current pulses are significantly modified.

## 6. Discussion

The simplified models [23,24], based on Ramo's expression for the drift current, are attractive as they provide a simple analytical description of the detector signals. However, the analytical expressions can only be obtained for the simplest approximations. The analytical form of the correlated drift (Ramo's-type) current for the junction type detectors is only applicable for a primary estimation of a transient shape. Different regimes in the formation of the pulsed response of detectors can appear in a real measurement. The time-dependent variations of the current transients may be determined by the injected charge dissipation through the domain drift, dielectric relaxation (due to media polarization effects), through carrier capture and thermal release processes in the traps containing material, via ambipolar diffusion processes. Several specific aspects of these phenomena have been discussed above.

### 6.1. Limitations of Models

Adaptability of the simplified models presented above is additionally limited by several factors. A principal limitation leads to the threshold values of the acquired drift velocity that should be significantly less than those of the electric field (light) propagation velocity in the material under consideration, to ensure the validity of the electrostatic approach. This condition excludes the possibility to detect the primary charged particles (moving with relativistic velocities) within the inter-electrode spacing. Thus, the secondary particle (the electron-hole pairs with a zero initial drift velocity at the injection point) induced currents should be calibrated to the primary particle impact. The specific feature of the prevailing drift current caused by the monopolar charge domain is the increase of the drift current with time within the vertex of a current pulse. To separate the neutral domain (locally generated) into the drifting charge sub-domains, the sensitivity threshold for the applied voltage appears. This limitation leads to a condition of the elevated values of bias voltage, at least *U* > *U_bi_* for the partially depleted semiconductor detectors. An enhancement of the external voltage may lead to the carrier velocity saturation, which complicates the analysis of the drift velocity field: *v_dr_(X_e_)*. Values of the highest external voltages are also restricted by the necessity to exclude repeated and non-linear drift processes, —such as the photo-electric gain moderated by carrier trapping and the avalanche processes of the impact ionization or Pool-Frenkel effect.

### 6.2. Limitations in the Evaluation Precision of the Depletion Layer Boundary

In the analysis of the junction type detectors, the parabolic approach has been employed, which relates the applied voltage and the width of the depleted region, and it is routinely exploited in device physics [15,25]. This approach enables one to simplify the expressions for the electric field description, as discussed in [15]. But this approach limits the precision in evaluation of the characteristic widths of *w_0_, w_q_* and *w_FD_*. In the steady-state case, the Debye screening length *L_D_* can be a measure for the evaluation of the precision in the estimation of *w_0_*. Thus, the transition layer [15] width, expressed as:

(111) $$\lambda ={\left(\frac{2ɛ{ɛ}_{0}}{eN_{\text{Def}}}\right)}^{1/2}{U_{\lambda }}^{1/2,}$$

depends on a voltage drop *U_λ_*, ascribed to this transition layer, and on the effective doping of a material. Thereby, for the large resistivity material, a relative inaccuracy (*λ/w_0_*) of the determination of the effective widths *w_0,q,FD_* ± *λ* can be unacceptable in the range of small reverse bias voltages. Traps and their filling processes can also be a reason for the instantaneous and local changes of the effective doping. Thereby, the resolution limit in time scale is expressed through the relaxation times as:

(112) $$\tau_{M,\text{Ndef}}=\frac{\tau_{\text{TOF},U_{\lambda }}}{2}$$

with *τ_TOF, Uλ_* = *λ^2^/μ_e_U_λ_*. The latter condition is determined by a necessity to stabilize the geometrical boundary by the balance of the local fields of carrier diffusion and drift.

The transitional layers are actually inherent to the boundaries between the metallic electrodes and dielectric or external heavy doped layers of junctions. Owing to a short dielectric relaxation in the heavy doped layers, the semiconductor junction is preferential relative to a dielectric in between of electrodes.

### 6.3. The Impact of the Injected Charge Density

In the Ramo's derivation of the charge drift current, it was clearly proved the reciprocity principle: the reversibility and equality of the mutual action and reaction of the charged electrode and drifting charge. This is based on the conservation of the charge (*σ* induced electro-statically on the electrode plane by a drifting injected charge *q* = *δσ*), charge momentum *qdΨ/dt* (*qv_dr_*) and the electrostatic energy (*qΦ* = *σ U*). Here, *Φ* is the surface equi-potential surrounding the drifting charge. The main equation for the current density (for instance, considering the motion of the electron domain) can be directly obtained by using the electrostatic energy balance *δqΦ* = −*δ(σU)*. Here, *δ* means a change in the electrostatic energy due to a variation of the surface charge (*δσ*) on the electrode, which should be balanced by a change in the energy of the moving charge *q_e_*. In the latter balance, *q_e_* is assumed to be invariable, and these energy changes are ascribed to the changes of the potential *δΦ*(*X*_e_), —during charge drift. The temporal changes of the surface charge on the electrode gives current density variations dependent on time (for a fixed external voltage), and this current density is generally expressed as *j(t)* = *dσ/dt* = −(*q_e_/U)(dΦ/dX_e_)(dX_e_/dt*). Accepting the general electrostatic relation E= –grad*Φ* = n_e_*q_e_/εε_0_* = E_q_ and assuming that instantaneous *E_q_*(*Ψ_e_*) = *q_e_Ψ_e_*/*εε*_0_, for its scalar representation, the expression for the current density is rearranged as *j(t)* = *dσ/dt* = *q_e_(d^2^/U)(q_e_/dεε_0_)[d(X_e_/d)/dt]* = *q_e_(τ_TOF,e_/τ_M,q_)[d(X_e_/d)/dt]*. Thereby, the just derived current density (on the basis of electrostatic energy conservation in the case of our consideration) is consistent with Ramo's derivation (also made on the basis of energy conservation) if *τ_TOF,e_/τ_M,q_* = 1. On the other hand, the equality of *τ_TOF,e_* and *τ_M,q_* is consistent with electrostatic induction approach. Using the scalar values of the field within the inter-electrode space as *E_σ_* = *U*/*d* and divE_q_ ≡ ▽·E_q_*(Ψ_q_)* = *q_e_*/*εε*_0_*d*, for the injected charge field *E_q_* (over a geometrical width *d)*, and a balance of electric fields and *E_σ_* = *U*/*d* = divE_q_ ≡ ▽·E_q_*(Ψ_q_)* = *q_e_*/*εε*_0_*d*, one gets a weighting field *W_E_* = divE_q_*/E_σ_* = *d*^−1^. This result validates the equal action and re-action of the surface (*σ* and *q*) charges and leads to the equality of the response times *τ_Mq,e_* = *τ_TOF,e_*. Actually, the equality of the response times *τ_Mq,e_*= *τ_TOF,e_* is ensured due to correlated changes of the acting voltage which is varied with *Ψ(t)*. To find the drift velocity field *v_dr_*(*X*_e_) = *dX*_e_/*dt*, the problem should be solved by consideration of the fields and charges in details.

As it has been demonstrated above, the drift velocity *v_dr_* is a function of the instantaneous charge domain position and the characteristic times: *τ_TOF,e_, τ_M,q_* and *τ_M,Ndef_*. It can be proved that the current density *j(t)* = 9*εε_0_μU^2^/*8*d^3^* obeys Mott-Gurney's law [26,27], for *v_dr_*(*X*_e_ = *d*/2) = *μ* [*(U/d)* + *(q_e_/*2*εε_0_)*]. Therefore, all the applied voltage *U* drops within the gap between the high potential electrode and the *q_e_* domain, during the initial phase of a drift process. The drifting domain additionally acts as a voltage sharing element (divider) with the parabolic-like characteristic of *U_2_* = *(*1−*Ψ^2^)U*. As a consequence, the drifting charge coordinate *Ψ(t)* ∼ exp(*t/τ_Mq_*) increases exponentially with the drift time *t*, leading to a variable drift velocity *v_dr_* ∼ *dΨ(t)/dt*) ∼ (*d/τ_Mq_)*exp(*t/τ_Mq_*) and the acceleration *a(t)* ∼ *(d/τ_Mq_^2^)*exp(*t/τ_Mq_*), to hold the processes (of drift, voltage sharing, and the induced amount of charge) correlated in time. In the case of the pure bipolar domain, the correlated drift of *q_e_* and *q_h_* sub-domains is equivalent to a widening of the quasi-neutral e-h domain.

In real detectors, the prevailing regime is the detection and collection of a small drifting charge, where *τ_Mq,e_*/*τ_TOF,e_* ≫ 1. Unfortunately, this regime can only be approximately considered within the one-dimensional approach. The reason is a slow dielectric relaxation (*τ_Mq,e_*/*τ_TOF,e_* > 1) of a small drifting charge *q_e_*. Due to the small *q_e_*, the charge domain surface becomes corrugated under the action of the electrode charge (at boundaries of the detector electrode of finite surface area *S*) and the charge density gradients within the surface plane of a drifting domain. To stabilize the gradients (or the oblique action of *σ* surface segments), the drifting charge should vary its position in all three spatial dimensions. Thus, the lateral fields should be taken into account, —the charge of dangling bonds on perpendicular boundaries acting as the surface recombination sinks can be a reason for such fields. On the basis of the Lagrange variational principle, it can be understood that charge movements within both the electrode and the domain planes should be correlated, to react most rapidly on each others changes. Then, energy conservation can only be considered by an analysis of the three dimensional charge drift and diffusion problem. This leads to the appearance of charges and their neutralization currents on the perpendicular (to an inter-electrode drift direction) boundary planes of the base region of the junction type detector. The small drifting charge *q_e_* is able to terminate the electric field of the electrode's equal to its amount. Therefore, a small drifting charge acts as a linear voltage divider within the inter-electrode gap, and, consequently, the I-V characteristic obeys Ohm's law. Then, due to the charge drift, the acting voltage is different from that applied on electrode *U*, and it changes under the variation of the charge position. The small charge drift current density is less (in comparison with the regime of the large charge drift) due to the less effective voltage *U_ef_*, acting on the injected charge, *q_e_*. This leads to the increased *τ_TOF,e_(t)*. The approximation models for *U_ef_* should be applied to cover the entire range of the injected charge values. Namely, for an electron domain the ratio *R_e1_* = *τ_Mq,e_/τ_TOF,e_* and *R_e2_* =*τ_Mq,e_/τ_M,Ndef_* should be replaced by *R_ef,e1_* = *(τ_Mq,e_/τ_TOF,e_)(U_ef,e_/(U* + *U_FD_))* and *R_ef,e2_* = *(τ_Mq,e_/τ_M,Ndef_)(U_ef,e_/(U* + *U_FD_))* (which modifies the voltage) using the approximation *U_ef,e_* = 1.225q_e_d(Ψ_e_*^0^)^1/2^*/εε_0_*. Here, Ψ_e_*^0^ is the dimensionless location of a drifting domain at the end of a bipolar drift. While, for a hole domain drift, these are *R_ef,h1_* = (*τ_Mq,h_*/*τ_TOF,h_*)(*U_ef,h_/(U − U_FD_))* and *R_ef,h2_* = (*τ_Mq,h_*/*τ_M,Ndef_*)(*U_ef,h_/(U − U_FD_))* (which modifies the voltage) using the approximation *U_C,ef,h_* = 1.995q_h_d(Ψ_h_*^0^)^1/3^*/εε_0_*. These approximations can be understood by an equal redistribution between degrees of freedom for the three-dimensional motion, if *R_e,h_* ≠ 1. The applicability of the *U_ef_* approximation models has been verified by their relevance to stitch together the one-dimensional solutions of the bipolar and the monopolar drift, thereby matching the synchronous conservation of the charge, charge momentum and current density continuity. This enables one to get continuous current density variations within the simulated vertex of the charge drift current pulse.

### 6.4. Correlation with Experimental Results

As mentioned above, a vast variety of possible pulsed current transients, composed of drift, diffusion and displacement current components exists depending on the detector design and different external factors, such as the injected charge quantity, applied voltages, presence of traps, *etc.*

**Figure 3.**
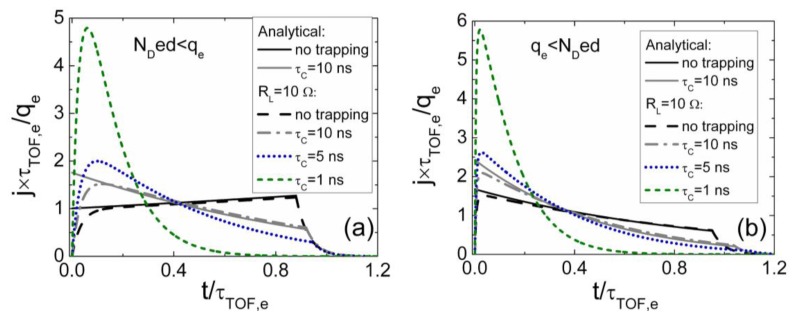
Transients of the normalized current density *j* × *τ_TOF,e_/q_e_* simulated for the large (**a**) and small (**b**) monopolar charge drift, simulated using different values of the carrier capture lifetime *τ_C_*. Solid curves are calculated using the analytical expressions, while the intermittent curves are obtained including the RC of a signal recording circuit.

**Figure 4.**
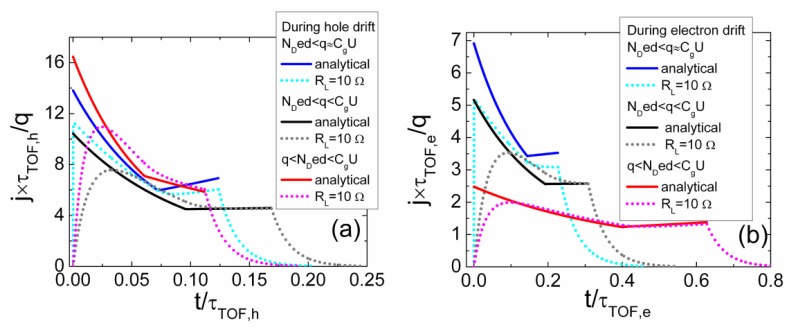
Transients of the normalized current density *j*× *τ_TOF,e_/q_e_* simulated for the bipolar charge drift during hole (**a**) and electron (**b**) drift times for various regimes, dependent on the densities of the drifting charge and of the space charge of ions. Solid curves are calculated using the derived above analytical expressions, while the dotted curves are obtained including the RC of a signal recording circuit.

The simulated specific transient shapes associated with different regimes of the injected charge drift are illustrated in Figures 3 and 4. These simulations have been performed by using the above presented models and keeping nearly the same charge drift conditions, while varying external voltage to implement the partial or full depletion regimes. The described analytical solutions enable ones to get the continuous curves of the drifting charge velocity and of current density as a function of a domain position and of time. These illustrations demonstrate that current density transients, containing a rather flat pulse vertex, can be found in experiments implemented for the small injected charge density by using a small load resistance. However, only the largest current components can be resolved due to a weak signal. The double peak containing current transient shape should be observed as usual in the case of the relative large charge drift, Ramo's type, regime. Thereby, depending on the applied load resistor and voltage, a vast variety of current transient shapes can be obtained in modelling of detector responses.

**Figure 5.**
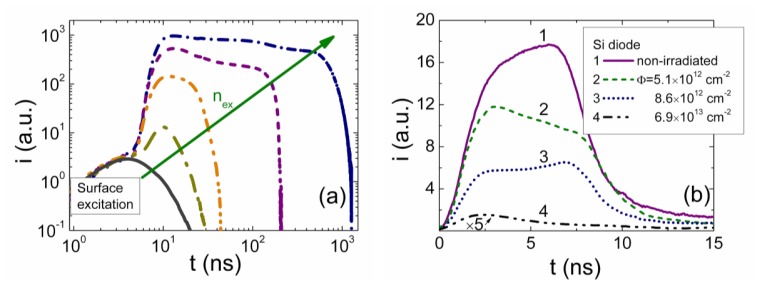
(**a**) Variation of current transients, registered in Si pad-detector biased with a fixed reverse voltage of value *U* ≤ *U_FD_*, as a function of the injected charge density. (**b**) The current transients, ascribed to the charge domain injected by a laser pulse, as a function of 25 MeV neutron irradiation fluence, evaluated from the exposure time of the in situ experiment.

Variations of current transients due to the injected charge drift, observed in experiments, are illustrated in Figures 5. These variations are demonstrated as a function of the injected charge density, in Figure 5a. These transients were recorded on the non-irradiated CERN standard pad-detectors, made of pure Si of 2 kΩ cm resistivity p^+^nn^+^ structures. The reverse bias voltage was kept fixed with the rather moderate values *U* < *U_FD_*. The surface domain was injected by strongly absorbed light laser pulse of 400 ps duration. Density of the injected domain of electrons, initially located nearby a junction boundary, was varied by changing intensity of excitation laser beam. The laser pulse was sufficiently shorter than *RC* ≅ 1 ns of the measurement system. The pulsed current was detected on 50 Ω load resistor and registered by a 1 GHz band real time digital oscilloscope.

The transients, —characteristic to the pulse durations controlled by the ambipolar diffusion lifetime, are illustrated in Figure 5a using a logarithmic time scale. Here, the pulse duration is varied in the time scale from a few of nanosecond, —that is inherent for the electrons drift time in the base region, to a few microsecond of the diffusion time. It can be easily noticed that current increases with time within a vertex of a pulse for the smallest injected charge densities, as predicted by models described. The current pulse has nearly exponential relaxation component, after domain reaches the rear electrode. Duration of the pulse vertex, measured between the initial (which is on the left in this scale) and rearward kinks within current transient is well correlated with electron drift time in *d* = 300 μm thick Si layer, using *μ_e_* ≅ 1,220 cm^2^/Vs value of the electron mobility. The enhancement of the injected charge density, proportional to the *n_ex_*_0_ injected carrier concentration, leads to the increasing delay (of the rear kink in current transient) and to an increase of the current (proportional to *q_e_*), approaching to the ambipolar diffusion lifetime *τ_D_* = *d^2^*/π^2^*D_a_*, ascribed to the main decay mode. The extracted value of the *D_a_* ≅ 15 cm^2^/s using the measured *τ_D_* time is in good agreement with parameters ascribed to a rather pure Si material.

Variation of current transients measured at extremely small excitation densities (close to those possible to detect at a threshold sensitivity of the measurement system equipped with proper current amplifier) is illustrated in Figure 5b. These transients were recorded during 25 MeV neutron irradiation when density of radiation induced traps varied with neutrons fluence. The electrical (*U_r_* = 150V) and optical (*n_ex0_*) parameters were carefully controlled to be fixed within measurements. The sample was kept in air just behind the neutron beam cone, while other experiment details are published in [14,28]. Evolution of the current transients is illustrated in Figure 5b, where currents had been controlled starting from that registered in the non-irradiated diode up to exposure duration for which the collected irradiation fluence reaches value of > 10^14^ n/cm^2^. The transient waveform inherent to the drift dominated current (curve 1 in Figure 5b) is observed in the non-irradiated diode, which is coincident with modelled transient shape. The radiation introduced traps determine a rapid reduction of carrier lifetime and an enhancement of carrier capture and generation current components. The transient of the carrier capture dominated current contains the single peak, and the relaxation-type curve occurs (curve 4 in Figure 5b). In the range of the intermediate exposure time (curves 2 and 3 in Figure 5b), the current pulse duration sustains values of the electron drift time within a diode base. The double peak and rising pulse vertex shapes alternate during increase of fluence. Applying of the smallest possible densities of the surface charge was sufficient to hide the inhomogeneities of the photo-generated domain. This evolution of current transients can be explained by the competition of carrier capture/generation and drift currents. For the largest irradiation fluence, the carrier trapping process becomes dominant, while a drift current component is completely competed, and the relaxation-type current pulse is only observed.

## 7. Summary

The models of the formation of the injected charge pulsed currents have been developed concerning the junction-type detectors. The partial and full depletion regimes have been analyzed. It has been shown, that, in junction detector, the drift time for the rather small density of the injected charge is shortened relatively to that of the capacitor-like detectors when a proper frame of reference (for comparison) is accepted and the characteristic relaxation times are matched. The description of the current pulse shape for the large injected charge drift in a finite area detector is coincident with that derived for the correlated drift (Ramo's-type) expressions. However, the induced currents obtained for the regimes of the small injected charge and of partial depletion lead to deviations from the Ramo's expressions. The analysis of the drift velocity field revealed the current increase within a vertex of the current pulse, for the monopolar drift regime. It has been shown, that presence of carrier traps considerably modifies the shape of the current transients. For the extremely large density of the injected charge *q* > *C_g_U*, the ambipolar diffusion of the injected carriers may become dominant in formation of the injected charge current pulse. It has been illustrated, that synchronous action of carrier drift, trapping, generation and diffusion lead to a vast variety of possible current pulse waveforms. Experimental illustrations of the current waveform variations obtained for both the rather small and the large charge density of the photo-injected domains are presented, based on study of Si detectors.
